# The Use of Biomarkers to Justify the Choice of the Proper Biologic Agent for the Treatment of Chronic Rhinosinusitis with Nasal Polyps: A Systematic Review

**DOI:** 10.3390/medicina62061188

**Published:** 2026-06-18

**Authors:** Georgios X. Papacharalampous, Theodora-Eleftheria Deftereou, Konstantinos Chaidas, Petros V. Vlastarakos, Jannis Constantinidis, Michael Katotomichelakis

**Affiliations:** 1 ORL Department, University Hospital of Alexandroupolis, 68100 Alexandroupolis, Greece; 2 Laboratory of Histology-Embryology, Medical School, Democritus University of Thrace, 68100 Alexandroupolis, Greece; tdeftera@med.duth.gr; 3 Department of Otorhinolaryngology, Medical School, Democritus University of Thrace, 68100 Alexandroupolis, Greece; konstantinoschaidas@duth.gr (K.C.); mkatotom@med.duth.gr (M.K.); 4 2nd University Department of Otorhinolaryngology—Head and Neck Surgery, Attikon University Hospital, 12461 Athens, Greece; pevlast@hotmail.com; 5 1st Department of Otorhinolaryngology, Medical School, Faculty of Health Sciences, Aristotle University of Thessaloniki, 54124 Thessaloniki, Greece; janconst@otenet.gr

**Keywords:** chronic rhinosinusitis with nasal polyps (CRSwNP), biomarkers, biologics, omalizunab, mepolizumab, dupilumab, benralizumab, reslizumab, tezepelumab

## Abstract

*Background and Objectives:* Chronic rhinosinusitis with nasal polyps (CRSwNP) is a heterogeneous type 2 inflammatory disease for which biologic therapies have expanded treatment options; however, biomarkers capable of guiding biologic selection remain poorly defined. This systematic review aimed to evaluate the available evidence regarding predictive and prognostic biomarkers associated with currently available biologic agents for CRSwNP (omalizumab, dupilumab, mepolizumab, benralizumab, reslizumab, and tezepelumab). *Materials and Methods:* A systematic search of PubMed/MEDLINE, Embase, Google Scholar, and the Cochrane Library identified studies published between January 2006 and September 2025. *Results:* Twenty-five eligible studies, including 12 randomized controlled trials, 12 systematic reviews/meta-analyses, and one indirect treatment comparison study, were analyzed. Multiple biomarkers, including blood eosinophils, total IgE, periostin, eotaxins, eosinophil cationic protein, IL-5, TARC, PARC, and urinary leukotriene E4, were evaluated across biologics targeting IgE, IL-4/IL-13, and IL-5 pathways. *Conclusions:* Although several biomarkers reflected the modulation of type 2 inflammation and disease activity, no validated biomarker has reliably predicted the superiority of one biologic over another. Nasal IL-5 showed potential for predicting the response to anti-IL-5 therapy but requires further validation. Current evidence supports biomarker use primarily for confirming type 2 inflammation rather than guiding biologic selection. Prospective biomarker-driven and head-to-head comparative studies are needed to enable precision medicine approaches in CRSwNP.

## 1. Introduction

During the last twenty years, relevant scientific research has been conducted with regard to the development of biologics, which would provide clinicians with new treatment options towards precision medicine concepts in chronic rhinosinusitis (CRS). Existing literature supports that up to 40% of CRS patients do not respond adequately to standard medical treatment that mainly involves systemic corticosteroids and/or intranasal corticosteroids [1,2].

At present, randomized double blind, placebo-controlled trials have already been completed, and useful results and conclusions have been published so far. Inclusion criteria usually involve evidence of type 2 inflammation, the need for systemic corticosteroids or contraindication to systemic steroids, significantly impaired quality of life, and/or the significant loss of smell and/or a diagnosis of comorbid asthma.

However, given the heterogeneity of the studies, the use of several new molecules and the different research protocols involved, direct comparisons among these studies and biologic agents remain extremely difficult. Moreover, a recent literature search performed by us demonstrated that there are still no head-to-head clinical trials available to compare such biologic treatments.

Although any patient considered eligible to receive any of the current biologic drugs for CRSwNP needs to suffer from type 2 CRSwNP, which can be demonstrated by a former biopsy revealing tissue eosinophilia or suspected based on comorbid asthma or other type 2 disease in the same patient [2], no definite evidence-based criteria (such as biomarkers) can assist clinicians in selecting the most appropriate biologic for individual patients with inadequately controlled CRSwNP. As dupilumab has been demonstrated to be the most efficacious biologic drug for CRSwNP disease and is well tolerated, about 85% of patients in Europe are currently treated with dupilumab. However, financial aspects, especially for long-term treatment schedules, and switching among biological drugs for achieving and maintaining a clinical response need also to be taken into account, and more knowledge needs to be accumulated in currently performed registries [3]. The aim of the present study is to summarize and review the knowledge gained regarding the use of possible biological markers (biomarkers), which are currently involved to justify the selection of the proper biologic agent for CRSwNP treatment. All eligible RCTs, systematic reviews, and indirect treatment comparison studies assessing potential biomarkers for predicting the efficacy of omalizumab, mepolizumab, dubilumab, benralizumab, and reslizumab were analyzed and critically reviewed, focusing on all available relevant endpoints and outcomes assessed by the researchers.

This is the first attempt of a systematic review of the current literature that exclusively covers the challenging issue of investigating/identifying possible biomarkers with potential predictive/prognostic value on the efficacy of the specific biologic agents. This particular attempt focused on two main aspects of the issue studied: on finding the biological markers associated with the satisfactory efficacy of biologic agents and/or on finding the biomarkers associated with poor responses to specific biologic treatment regimens.

## 2. Materials and Methods

This study was conducted as a systematic review and meta-analysis in accordance with the Supplementary Material PRISMA 2020 Checklist [4].

An extensive search in Pubmed/Medline, Embase, Google Scholar, and Cochrane Database/Library was conducted via the following searching string: “omalizumab AND/OR mepolizumab AND/OR dupilumab AND/OR reslizumab AND/OR benralizumab AND/OR biologics AND/OR biomarkers AND chronic rhinosinusitis with nasal polyps AND/OR CRSwNP”.

Non-human studies, as well as papers written in other languages and not accompanied by an English summary/abstract, were excluded.

All papers published between January 2006 and September 2025, with an English abstract, were included in the initial stage of this study. All papers were initially evaluated based on the title and abstract. The final number of initially identified papers was 291 (n = 291).

Eligibility criteria for further paper screening were as follows: studies/papers with full text in English, adult studies, CRS-relevant studies, clearly described intervention or research protocol, and specific biologic agent(s) assessed, as well as adequately analyzed primary and secondary endpoints–outcomes and clearly reported conclusive data regarding specific biomarkers with possible predictive/prognostic value on the efficacy of biologics for the treatment of CRSwNP.

We thoroughly examined the full copy of any paper deemed eligible and reviewed the bibliographical references for additional potentially relevant papers. The eligibility of studies was assessed by two independent reviewers, and any conflicts were resolved through consensus.

From the initially identified 291 records, the authors/reviewers excluded 213 papers due to apparently irrelevant content for one or more of the following reasons: non CRS-relevant studies, no endpoints mentioned at all, biologic agents assessed were not omalizumab or mepolizumab or dupilumab or benralizumab, or reslizumab, no biomarkers clearly assessed as possible prognostic/predictive factors for treatment efficacy.

Papers sought for retrieval numbered 78. From those 78 published papers, 31 more were excluded from the present study, because the full text was not available at all or it was not available in the English language (12 papers) or the provided data regarding biomarkers such as endpoints, outcomes, or other crucial parameters were found to be inadequate or unclear (19 papers).

The full text of the remaining 37 eligible papers was then read. Twelve more papers were also excluded because relevant data presented and analyzed mainly referred to other comorbidities (e.g., asthma, aspirin-exacerbated respiratory disease, etc.) and/or those studies assessed several biomarkers that express the pathophysiology of the disease (CRSwNP) regardless of the therapeutic use and efficacy of biologic agents.

**Figure 1:**
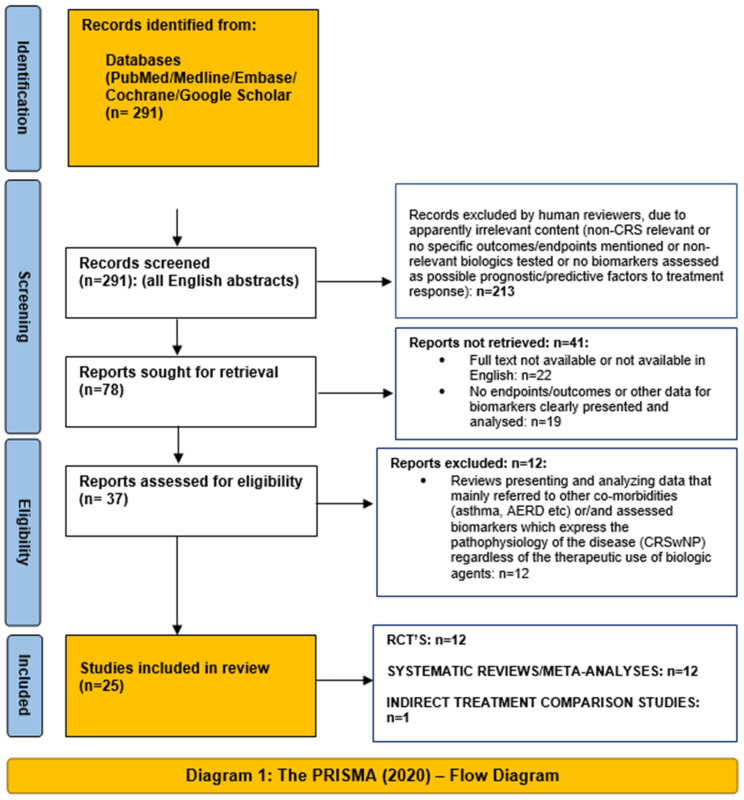
The PRISMA flow diagram. (PRISMA: Preferred Reporting Items for Systematic Reviews and Meta-Analyses.)

The final number of studies/published papers, finally included in the present analysis, was restricted to 25: 12 RCTs, 12 systematic reviews/meta-analyses, and one Indirect Comparison Treatment study (ICT) (Figure 1, Prisma Flow Diagram).

## 3. Results

### 3.1. Data from Randomized Controlled Trials

Twelve randomized controlled studies (12 RCTs), completed and published between 2006 and 2022, were finally included in the present analysis [5,6,7,8,9,10,11,12,13,14,15,16].

As far as those RCTs are concerned, the vast majority were either the original randomized, double-blind, multicenter, placebo-controlled Phase 3 trials in the field (POLYP 1 & 2 [8], SINUS-24 & SINUS 52 [7], SYNAPSE [10], OSTRO [15]) and smaller original RCTs or (open-label) extension studies, sub-group analyses, and responder analyses that used pooled data from the above-mentioned original Phase-3 studies.

Three (3) of the included studies assessed omalizumab and its effects in the treatment of CRS, along with possible relevant predictive/prognostic biomarkers [8,9,16], whereas dupilumab and its biomarkers were evaluated in five studies [6,7,12,13,14], mepolizumab and its biomarkers in two studies [10,11], benralizumab and biomarkers in one study [15] and reslizumab and biomarkers in one study [5].

The scheduled follow-up time among all 12 studies varied between 16, 24, and 52 weeks.

The primary outcomes and endpoints analyzed in most studies were as follows: change from baseline in the total endoscopic polyp score (NPS) and nasal congestion severity score (NCS), as well as changes in the Lund–Mackay CT score (LMK). Other parameters also assessed (either as primary or secondary outcomes) were the change from baseline in sense of smell and nasal discharge, the need for surgery, and/or systemic steroids. Other measures used to assess the outcomes were NPS, NCS, SNOT-22, VAS-symptom score, UPSIT, Lund–Mackay CT Score, PNIF, FEV, FVC, PEFR, AQLQ, and TNSS.

Potential prognostic biomarkers evaluated were blood eosinophil and basophile counts, serum total IgE, plasma concentrations of thymus and activation regulated chemokine (TARC), periostin, eotaxin-3, eosinophil cationic protein (ECP), serum tryptase, serum thymus and activation regulated cytokine, serum PGD_2_ and 24 h urinary leukotriene E_4_ (LTE_4_), and total IgE and ECP in nasal secretions, along with ECP, total IgE, IL-4, IL-5, IL-17, TNF-a, IL-10, IL-1b, IL-6, IL-13, TARC, eotaxin-1, eotaxin-2, eotaxin-3, and PARC in tissue homogenates and eosinophils in nasal lavage.

All 12 studies are presented in full detail in Table S1, including the study ID, the contributors and the aim of each study, the type of research protocol, the number of patients participated, the treatment regimen given, the follow-up time, and the outcomes/conclusions focusing on possible predictive/prognostic biomarkers assessed (Table 1).

**Biomarkers for omalizumab:** serum total IgE levels and eosinophils in nasal lavage were evaluated in the three RCTs included in the present analysis [8,9,16]. Serum IgE levels were only measured as baseline data for patient selection and recruitment rather than as a potential predictive biomarker. Omalizumab generally decreased the mucosal concentration of IgE, as well as the tissue eosinophil cell density. As far as eosinophils in nasal lavage are concerned, there were no differences in net change between the treated groups (omalizumab-treated patients vs placebo-treated).

**Biomarkers for dupilumab: **blood eosinophil count, serum total IgE, thymus and activation regulated chemokine (TARC), plasma periostin, plasma eotaxin-3, eosinophil cationic protein (ECP) concentrations and total IgE in nasal secretions, serum PGD_2_, and 24 h urinary leukotriene E_4_ (LTE_4_) were assessed [6,7,12,13,14]. Dupilumab treatment reduced multiple biomarkers of type-2 inflammation in both nasal secretions and polyp tissues in patients with CRSwNP. The antagonism of IL-4Ra signaling suppresses IL-4/IL-13-dependent processes, such as mucosal IgE formation and the expression of chemokines. Blood eosinophil levels may not be a suitable marker for dupilumab efficacy in CRSwNP.

**Biomarkers for mepolizumab:** all included RCTs assessed blood eosinophil counts [10,11]. Serum IL-5Ra, nasal IL-5Ra and serum ECP were assessed in a few studies (not included in the present analysis). The use of mepolizumab significantly decreased blood eosinophil counts compared with the placebo. No rebound eosinophilia was observed in the relevant RCTs included in the present study.

**Biomarkers for reslizumab:** peripheral blood eosinophil counts and peripheral blood and nasal secretions of IL-3, IL-5, SOL IL-1Ra, eotaxins-2, 3, ECP, and granulocyte-macrophage colony-stimulating factor (GM-CSF) were assessed [5]. Blood eosinophil numbers and concentrations of eosinophil cationic protein were reduced up to 8 weeks after treatment in serum and nasal secretions. Responders had increased IL-5 concentrations in nasal secretions at baseline compared with non-responders. Logistic regression analysis revealed that increased nasal IL-5 levels (>40 pg/mL) might be used to predict the response to anti-IL-5 treatment.

**Biomarkers for benralizumab: **blood eosinophils and basophil counts were assessed [15]. As expected, blood eosinophils were nearly completely depleted, and basophil counts were reduced in the benralizumab-treated group vs. placebo group. Subgroup analyses suggested influences of comorbid asthma, number of polyp surgeries, gender, body mass index, and baseline blood eosinophils on the treatment effects. Table 1 summarizes all potential biomarkers assessed/proposed for each of the five biologics analyzed.

### 3.2. Data from Systematic Reviews/Meta-Analyses, Indirect Treatment Comparison Studies (ITCs), and Reviews

Thirteen (13) studies [17,18,19,20,21,22,23,24,25,26,27,28,29], completed and published between 2015 and January 2023, were finally included in the present analysis: three (3) systematic reviews [17,18,19], one (1) Indirect Treatment Comparison study [20], and nine (9) reviews [21,22,23,24,25,26,27,28,29].

Two of the included systematic reviews assessed possible biomarkers for omalizumab, mepolizumab, dupilumab, and reslizumab. One systematic review focused on the investigation of possible biomarkers for omalizumab, dupilumab, mepolizumab, benralizumab, and tralokinumab.

The only Indirect Treatment Comparison Study included focused on identifying possible predictive biomarkers for omalizumab and dupilumab, based on data from POLYP-1, 2 and SINUS-24, 52 RCTs studies, respectively [20].

The follow-up time varied between 16, 24, and 52 weeks among all those studies.

The main measures commonly used to assess the outcomes of biologics, in terms of efficacy, were the Total Nasal Endoscopic Polyp Score (NPS), CT score, Quality of life measures [SNOT-20, SNOT-22, SF-36, Rhinosinusitis Outcome Measurement Instrument (RSOM-31)], Nasal Airflow (PNIF), and Olfaction (via UPSIT).

Possible biomarkers assessed for all biologics in the majority of included studies were as follows: blood eosinophil and basophile counts, eosinophilic cationic protein, secreted Il-5a, total serum IgE, plasma eotaxin-2, eotaxin-3, IL-6, IL-1β, IL-4, IL-5, IL-10, IL-17, IL-25, IL-33, TNF-α, TARC, periostin, nasal matrix metallopeptidase-9 and myeloperoxidase, NP levels of CCL24, CCL26, ECP, IL-5, and IgE, pulmonary and activation-regulated chemokine, urinary levels of leukotriene E4 (LTE4), and prostaglandin D2 metabolite 9a,11b- prostaglandin F2 (PGD2M).

All 13 systematic reviews/meta-analyses, the ITC, and reviews included in the present study are listed in Table S2 in full detail. This table contains data regarding the contributors and year of publication, the aim of each study and the parameters/outcomes assessed, the type of review/analysis (systematic review/meta-analysis/ITC, review), the trials/studies/patients included, and the outcomes in brief (Table 2).

**Biomarkers for omalizumab:** Patients with CRSwNP and asthma whose levels of IgE in the serum are at least 30 IU/mL can be treated with omalizumab. However, patients with IgE levels less than 76 IU/mL do not seem to benefit significantly [28]. Omalizumab also reduced the mucosal concentration of IgE, as well as the tissue eosinophil cell density.

Treatment with omalizumab for 12 months [30] induced significant reductions in urinary levels of leukotriene E4 (LTE4) and the prostaglandin D2 metabolite 9a, 11b-prostaglandinF2 (PGD2M), both markers of mast cell activation [26].

Periostin was confirmed to be decreased after treatment with omalizumab [21]. Omalizumab also reduced ECP and soluble IL-5Ra levels but provided a limited effect on local IL-5 and blood eosinophil counts [21,22,23,24,25,26]. Omalizumab treatment did not lead to significant differences in eosinophils in nasal lavage [17].

**Biomarkers for dupilumab: **Dupilumab inhibits the IL-4Ra sub-unit and therefore inhibits the effect of IL-4 and IL-13. However, tissue IL-4 or L-13 are not measured routinely in laboratories [28].

Dupilumab is associated with a transient increase in blood eosinophil counts, as eosinophils cannot leave the blood into the inflamed tissue anymore; however, blood eosinophils return to pre-treatment baseline values within 52 weeks post-treatment initiation [20,21,28]. There was a reduction in tissue eosinophil counts, total serum IgE, and plasma eotaxin-3 levels when comparing dupilumab treatment with placebo. A statistically significant reduction in total serum IgE and plasma eotaxin-3 levels was also demonstrated [18].

Dupilumab improved clinical symptoms along with decreasing tissue levels of eotaxins 2 and 3 (also called CCL24 and CCL26: inflammatory chemokines that bind to CCR3-motif chemokine receptor-3, which is related to eosinophil trafficking and the Th2 immune response), ECP, IL-5, IgE, and pulmonary and activation-regulated chemokine (PARC) in nasal polyp tissue [21]. Serum total IgE suppression was gradual, with a greater effect related to a longer duration of therapy [7].

In another analysis of polyp tissue concentrations, total IgE, ECP, eotaxin-2, eotaxin-3, IL-13, and PARC were significantly lower in the dupilumab group by the end of treatment than in the placebo group, whereas IL-6, IL-1β, IL-4, eotaxin-1, IL-5, IL-10, IL-17, IL-33, TNF-α, or TARC was not significantly different between groups [19,20,27].

Measurements of nasal ECP, eotaxin-3, and total IgE were unique to the SINUS-52 study protocol.

**Biomarkers for mepolizumab: **The use of mepolizumab, as expected, led to a significant reduction in blood eosinophil counts compared to the placebo group, without rebound eosinophilia [17].

Patients receiving mepolizumab demonstrated a reduction in blood eosinophil counts from baseline from week 8 to week 25 [18]. There was no reduction in the placebo group [17,18].

Mepolizumab reduces eosinophil counts, nasal and peripheral IL-5, soluble IL-5Ra, and ECP levels in patients with CRSwNP [21]. Mepolizumab also reduces several other local and systemic markers of type-2 inflammation, such as IgE, periostin, nasal matrix metallopeptidase 9, and myeloperoxidase and decreases the numbers of circulating basophils and nasal levels of pro-inflammatory mediators such as prostaglandin D2, prostaglandin F2 alpha, leucotriene B4, and thomboxane in patients with CRSwNP and non-steroidal anti-inflammatory drug-exacerbated respiratory disease (N-ERD) [18,21].

**Biomarkers for reslizumab**: Reslizumab induced a sustainable reduction in blood eosinophil counts starting from 12 h after administration [18]. This returned to baseline at 12 weeks and showed a rebound increase thereafter, as reported in all patients in the 1 mg/kg group and in 4/6 patients in the 3 mg/kg group.

Serum eosinophilic cationic protein (ECP) and secreted IL-5α decreased with reslizumab compared with placebo groups for the first few weeks after treatment. In contrast, there was no change in plasma or nasal eotaxin levels. Reslizumab also reduced eosinophil counts, nasal and peripheral IL-5, soluble IL-5Ra, and ECP levels [18]. A single dose of IV reslizumab at 1 mg/kg or 3 mg/kg reduced the total nasal polyp score in half of the patients. Moreover, a responder analysis showed the increased baseline nasal secretion IL-5 levels in responders versus non-responders. Twelve weeks after the withdrawal of reslizumab, a deterioration in the nasal polyp score was demonstrated [9,26].

**Biomarkers for benralizumab**: Benralizumab reduced the tissue eosinophil cell density [19]. Benralizumab in addition to standard-of-care treatment for CRSwNP reduced the nasal polyp score, nasal blockage, and smell disorders, as well as eosinophil counts, in patients with CRSwNP [21,22,23].

It is of high interest that benralizumab is further investigated as an add-on treatment for CRSwNP because it also decreases basophil levels, which were shown to be elevated in nasal polyps and may play a significant role in the disease [29].

## 4. Discussion

### 4.1. The Current Biologics Involved in CRSwNP Treatment Along with Their Relevant Mechanisms of Action and Approval Status

Several biologic agents, targeting different aspects of type-2 inflammation, have already been applied in the treatment of CRSwNP, especially during the last decade (Table 2).

Omalizumab, distributed under the brand name “Xolair^®^”, is a biologic agent used to treat asthma, chronic rhinosinusitis with nasal polyps [8], and urticaria. Many clinical trials and case-series studies on omalizumab have been completed since 1996, and a large number of research reports have been published since 2000. In the USA, omalizumab is approved by the Food and Drug Administration (FDA) to treat moderate to severe persistent asthma (2003), chronic idiopathic urticaria, and CRS with nasal polyps (2020). Omalizumab has also been approved by the European Commission as an add-on treatment for CRSwNP since 2020 (Table 2).

Dupilumab, distributed under the brand name “Dupixent^®^”, is a monoclonal antibody blocking interleukin 4 and interleukin 13. Dupilumab is approved by the FDA for the treatment of moderate-to-severe atopic dermatitis (2017), moderate-to-severe asthma (2018), and chronic rhinosinusitis with nasal polyposis (2019) [7]. The agent has also been approved by the European Commission as an add-on treatment for CRSwNP since 2019 (Table 2).

Dupixent is indicated as add-on therapy, along with intranasal corticosteroids for the treatment of adults with severe CRSwNP, when systemic corticosteroids and/or surgery do not provide adequate disease control, in Europe (2019). Recently (on May 2022), the indication for dupilumab was extended to cover eosinophilic esophagitis in patients from 12 years of age and older, weighing at least 40 kg.

Mepolizumab, distributed under the brand name “Nucala^®^”, is a fully humanized monoclonal antibody that targets IL-5 and prevents binding to the IL-5 receptor on the surface of eosinophils. It is indicated as an add-on treatment for severe refractory eosinophilic asthma in adults, adolescents, and children aged 6 years and older (FDA-2015). It is also approved as an add-on therapy with intranasal corticosteroids for the treatment of adult patients with severe CRSwNP that is not adequately responding to therapy with systemic corticosteroids and/or surgery [10] (FDA-2021, European Commission 2021). Moreover, mepolizumab is also indicated as an add-on treatment for adult patients with inadequately controlled hyper-eosinophilic syndrome, when a non-hematologic secondary cause is not identified (FDA-2020).

Benralizumab, distributed under the name “Fazenra^®^”, is a humanized recombinant monoclonal antibody of the isotype IgG1k immunoglobulin that specifically binds to the alpha-chain of the interleukin 5 receptor (IL-5R) expressed on eosinophils and basophils. Benralizumab was approved by the FDA in November 2017 for the treatment of severe eosinophilic asthma. It was granted designation as an orphan drug by the FDA for the treatment of eosinophilic esophagitis in August 2019. At present, the agent is not approved by FDA/European Commission for use in CRSwNP, as it is still in Phase-2 trials.

Reslizumab, distributed under the names “Cinqair^®^” and “Cinqaero^®^”, is a humanized monoclonal antibody targeting human interleukin-5 (IL-5). Reslizumab binds specifically to IL-5, a cytokine responsible for the differentiation, maturation, recruitment, and activation of human eosinophils. By binding to human IL-5, it blocks its biological function; consequently, the survival and activity of eosinophils are reduced. The benefits related to reslizumab are its ability to reduce the exacerbation rate and improve lung function and asthma-related quality of life in patients with severe eosinophilic asthma (with blood eosinophil counts ≥ 400 cells/μL) and with at least one previous asthma exacerbation in the preceding year. In March 2016, the FDA approved reslizumab for use with other asthma medicines for the maintenance treatment of severe asthma in people aged 18 years and older. At present, the agent is not approved by the FDA/European Commission for use in CRSwNP, as it is still in Phase-3 trials.

### 4.2. Available Data Regarding Possible Predictive/Prognostic Biomarkers from Existing Original (Mother) Studies

Original (mother) studies assessing the efficacy of omalizumab, dupilumab, mepolizumab, benralizumab, and reslizumab vs. placebo for the treatment of CRSwNP are POLYP 1&2 [8], SINUS 24&52 [7], SYNAPSE [10], OSTRO [15], and Gevaert’s [5] (2006) original study. All those studies are listed in Table 2 and Table 3. As stated above, only omalizumab, dupilumab, and mepolizumab are currently approved by the FDA/European Commission for the treatment of CRSwNP (Table 3 and Table 4).

Interestingly, Phase-3 studies of omalizumab, dupilumab, and mepolizumab do not contain documented conclusive data regarding possible predictive/prognostic biomarkers for those specific biologics (Table 3). This fact obviously reflects the initial need for investigating the overall efficacy and safety of those agents when used as add-on therapy for CRSwNP rather than collecting and analyzing data for predictive biomarkers. As original studies did not focus on specific biomarkers, further analyses (via pooled data analysis, responder analyses or even extension-protocol studies, based on initial series) are still ongoing through RCTs, systematic reviews, or Indirect Treatment Comparison Studies.

As far as benralizumab is concerned, subgroup analyses of OSTRO-RCT’s data suggested possible influences of the baseline blood eosinophil count on treatment effects [15].

Gevaert el al. [5] demonstrated that blood eosinophil numbers and concentrations of eosinophil cationic protein were reduced up to 8 weeks after reslizumab treatment in both serum and nasal secretions. Responders showed increased IL-5 concentrations in nasal secretions at baseline compared with non-responders. Logistic regression analysis demonstrated that increased nasal IL-5 levels (>40 pg/mL) might predict the response to anti-IL-5 treatment.

### 4.3. Available Data Regarding Possible Predictive/Prognostic Biomarkers from Other Existing Recent RCT Studies and Systematic Reviews/Meta-Analyses

Jonstam et al. [6] (2019) reported that dupilumab treatment reduced multiple biomarkers of type-2 inflammation in nasal secretions and polyp tissues, in patients with CRSwNP. According to those researchers, this particular effect on the inflammatory process is based on the antagonism of IL-4Ra signaling, which suppressed IL-4/IL-13-dependent processes, such as mucosal IgE formation and the expression of chemokines.

Bachert et al. [7] (2019) presented the results of pre-specified exploratory analyses of biomarkers in dupilumab-treated patients with CRSwNP (SINUS-52 trial). In those analyses, dupilumab demonstrated a consistent decrease in concentrations of serum total IgE, periostin, TARC, and plasma eotaxin-3 at weeks 24 and 52 and an additional decrease in concentrations of ECP, total IgE, eotaxin-3, and IL-5 in nasal secretions at week 24. These authors also reported that dupilumab provided a non-statistically significant, transient increase in mean blood eosinophil counts (in both SINUS-24&52 studies). In the SINUS-52 study, blood eosinophil counts returned to baseline by the end of week 52.

Fujieda et al. [12] (2022-data from SINUS-52 study, in Japan) reported negative median percentage changes in blood biomarkers (eosinophils, total IgE, TARC, and periostin) in both dupilumab-treatment arms, by week 52. Smaller decreases were also demonstrated in the placebo-treated patients, with the only exception of periostin, which was increased by week 52.

Mustafa et al. [14] (2021) showed that dupilumab provided a significant decrease in fractional exhaled nitric oxide (FeNO), which is suggestive of decreased eosinophilic inflammation throughout the whole sino-pulmonary tract. The improvement in FeNO and other T2-related biomarkers was observed as early as four weeks after the initiation of dupilumab. The authors also stated that urinary leukotrienes, a key mediator of inflammation in aspirin-exacerbated respiratory disease-AERD (Leucotriene E_4_—LTE_4_), showed a prompt decrease after dupilumab treatment.

Hopkins et al. [13] (2021) published a post-hoc review based on data from SINUS-24 & 52 trials. They assessed biomarkers of type-2 inflammation from blood samples taken at study baseline. Those researchers concluded that patients, who had both more recent sinonasal surgeries and a higher number of such surgeries, are suffering from a high type-2 inflammatory burden, as reflected by the increased prevalence of type-2 inflammatory comorbidities (asthma, N-ERD) and greater baseline blood eosinophil counts and periostin levels, compared with non-operated patients. Dupilumab appeared to achieve the suppression of E4 leucotriene levels—an important biomarker of mast cell activation.

Tsetsos et al. [17] (2018) reported that, despite the significant decrease in eosinophils with the use of reslizumab, rebound eosinophilia appeared at week 24 and week 32 in 1 mg/kg and 3 mg/kg treatment groups, respectively. In contrast, the use of mepolizumab provided a significant reduction in blood eosinophils, compared with the placebo. No rebound eosinophilia was observed in the two RCTs assessing mepolizumab.

No statistically significant decrease in eosinophil counts was observed in dupilumab- or placebo-treated patients. The results were similar for omalizumab vs. placebo.

Transient increases in mean eosinophil counts were shown in dupilumab-treated patients with asthma (mean range across studies at baseline: 349–370 cells/μL; week 4: 515–578 cells/μL), CRSwNP (baseline: 440–448 cells/μL; week 16: 595 cells/μL), and atopic dermatitis (baseline: 434–600 cells/μL; week 4: 410–710 cells/μL), followed by a decline starting by week 24 to baseline or lower [12,17,31]. Dupilumab’s efficacy in chronic rhinosinusitis with nasal polyps from SINUS-52 appears to be unaffected by the eosinophilic status [12]. Overall, the blood eosinophil count is not considered to be a reliable biomarker for predicting dupilumab’s efficacy.

Serum IL-5Ra, nasal IL-5Ra, serum ECP, and nasal ECP were significantly decreased by reslizumab and mepolizumab (with the exception of nasal ECP) [17].

Iqbal et al. [18] (2020), in their systematic review, reported a reduction in eosinophil counts when comparing dupilumab plus mometasone furoate with placebo plus mometasone furoate. They also reported a statistically significant decrease in serum total IgE and plasma eotaxin-3 in dupilumab-treated patients vs. placebo-treated cases.

Patients receiving mepolizumab showed a reduction in blood eosinophil counts from baseline to week 25 (not observed in placebo-treated group) [11,18]. At week 8 after treatment with mepolizumab, the treatment group showed a significant reduction in blood eosinophils, serum ECP levels, and serum IL-5Ra levels [11,18].

Walter et al. [19] (2020), in a systematic review, concluded that dupilumab reduced the concentration of IL-13 but not IL-4. Moreover, dupilumab decreased mucosal eosinophil granule proteins.

Those authors also reported that omalizumab reduced the mucosal concentration of free IgE by providing IgE/anti-IgE complexes, which are not measurable, whereas omalizumab, mepolizumab, and benralizumab reduced the tissue eosinophil cell density.

Peters et al. [20], in their ITC and responder analysis, commented on the issue of possible biomarkers to justify the use of appropriate biologic agents for CRSwNP patients. They supported that the higher treatment effect size of dupilumab could be attributed to a broader spectrum of action on type-2 inflammation compared with omalizumab, as dupilumab targets IL-4 and IL-13, thus decreasing IgE, eotaxin-3, IL-5, tissue eosinophilia, and mast cell production of inflammatory mediators.

Wang et al. [28] found that the expression of periostin is related to IL-4 and IL-13 rather than to IL-5. Jonstam et al. [6] showed that IL-5 is positive in tissues when serum periostin is higher than 48.5 ng/mL (sensitivity 93.5%, specificity 62.5%). Periostin was confirmed to be decreased after treatment with mepolizumab and omalizumab.

All available conclusive data, for the five biologics assessed in the present analysis, are briefly summarized in the following Section 4.4.

### 4.4. Conclusive Data for Potential Predictive/Prognostic Biomarkers

#### 4.4.1. Omalizumab

The dosage and frequency of omalizumab administration are generally calculated based on the baseline total serum IgE and bodyweight. However, the baseline total serum IgE level does not actually predict tissue IgE concentrations and thus does not allow the response to be predicted. It seems that free IgE, rather than total IgE, is closely related to the response to omalizumab; however, it is difficult to monitor free serum or tissue IgE levels, specifically once omalizumab treatment is initiated [32]. The present indication for omalizumab includes patients with CRSwNP and asthma whose levels of serum IgE are at least 30 IU/mL. However, patients with IgE levels less than 76 IU/mL do not seem to benefit significantly, as stated in omalizumab’s spc. In a pooled analysis, add-on therapy with omalizumab resulted in meaningful treatment benefits in all four baseline IgE quartiles (IU/mL: 0–75, 76–147, 148–273, ≥274) across the range of outcome variables. However, efficacy was greater for more outcome variables (including exacerbations) in the upper three IgE quartiles, with similar benefits in each of these three [32]. Omalizumab also reduces the mucosal concentration of IgE, as well as tissue eosinophil cell density.

Treatment with omalizumab for 12 months induced significant reductions in urinary levels of leukotriene E4 and the prostaglandin D2 metabolite 9a, 11b- prostaglandin F2, both markers of mast cell activation. Although periostin (a multifunctional matricellular protein in inflammatory microenvironments) levels were reported to be decreased after treatment with omalizumab in asthma patients, this effect is apparent only when CRSwNP is not present. Conversely, periostin levels could be elevated in omalizumab-treated patients with CRSwNP. This finding probably reflects the fact that periostin production may be dependent on signaling pathways differing from the conventional atopic reactivity network. Omalizumab may interfere less with such pathways [33].

#### 4.4.2. Mepolizumab

The use of mepolizumab significantly decreased blood eosinophils compared with the placebo. No rebound eosinophilia was observed. Mepolizumab also reduced nasal and peripheral IL-5, soluble IL-5Ra, and ECP levels in patients with CRSwNP. Several other local and systemic biomarkers of type-2 inflammation were decreased: periostin, nasal matrix metallopeptidase-9 and myeloperoxidase, numbers of circulating basophils, and nasal levels of pro-inflammatory mediators such as prostaglandin D2, prostaglandin F2 alpha, leucotriene B4, and thomboxane in patients with CRSwNP and N-ERD. Mepolizumab did not significantly change IgE levels in treated patients. In contrast, some authors reported that the agent demonstrated a marginal effect on basophils [34,35].

#### 4.4.3. Dupilumab

Dupilumab is generally associated with a transient increase in blood eosinophil counts, which returns to the pre-treatment baseline by 52 weeks post-treatment initiation or earlier. There was a reduction in eosinophils when comparing dupilumab in combination with mometasone furoate nasal spray (MFNS) with the placebo/MFNS. Statistical significance was confirmed for total serum IgE and plasma eotaxin-3 levels.

At the end of treatment, in the analysis of polyp tissue, total IgE, ECP, eotaxin-2, eotaxin-3, IL-13, and PARC were significantly lower in the dupilumab group than in the placebo group.

Dupilumab can block the IL-4Ra sub-unit to inhibit the production of IL-4 and IL-13. The relatively higher treatment effect size of dupilumab, compared with the effect of omalizumab [36], might be attributed to a broader mechanism of action on type 2 inflammation compared with omalizumab, because dupilumab targets IL-4 and IL-13, which have been shown to decrease IgE production, as well as reduce other aspects of type 2 inflammation such as eotaxin-3, IL-5, tissue eosinophilia, and mast cell production of inflammatory mediators.

Although dupilumab has been shown to reduce a broader range of type 2 inflammatory biomarkers, including eosinophil-associated cytokines, IgE-related pathways, and markers of epithelial activation, the extent to which this translates to superior clinical outcomes or should influence biologic selection remains uncertain. Current treatment decisions are primarily guided by the clinical phenotype, comorbidities, biomarker profiles, and regulatory indications rather than by the breadth of biomarker modulation alone. Head-to-head comparative studies are lacking, and therefore, the broader anti-inflammatory effects of dupilumab should be interpreted as mechanistic observations rather than definitive evidence for preferential use.

#### 4.4.4. Benralizumab

Blood eosinophils were nearly completely depleted and basophil counts were reduced in the benralizumab-treated patients. Subgroup analyses suggested influences of co-morbid asthma, the number of NP surgeries, sex, body mass index, and baseline blood eosinophil counts on the treatment effects. Benralizumab also reduced the tissue eosinophil cell density. Interestingly, this particular agent also decreased basophil levels, which were shown to be elevated in nasal polyps. Those findings should be further investigated: although benralizumab leads to a near complete suppression of eosinophils, the drug only weakly suppresses the NP score. This means that the importance of eosinophils and thus benralizumab in CRSwNP needs to be questioned.

#### 4.4.5. Reslizumab

Blood eosinophil numbers and concentrations of eosinophil cationic protein were reduced up to 8 weeks after treatment in both serum and nasal secretions. Responders demonstrated increased IL-5 concentrations in nasal secretions at baseline, compared with non-responders, and logistic regression analysis also revealed that increased nasal IL-5 levels might predict the response to anti-IL-5 treatment.

Elevated nasal IL-5 has emerged as a potential predictor of the response to reslizumab because it reflects local eosinophilic inflammation, the primary target of IL-5 blockade. However, its application in routine clinical practice remains limited by the lack of standardized sampling techniques, validated assay thresholds, and widespread laboratory availability. Furthermore, the reproducibility and cost-effectiveness of nasal IL-5 measurements have not been established across diverse clinical settings. Consequently, while nasal IL-5 represents a promising biomarker for personalized therapy, further prospective validation and assay harmonization are required before it can be incorporated into routine treatment algorithms.

Although both benralizumab and reslizumab still lack official FDA and EMA licensing for this specific condition, there is promising scientific evidence in published literature supporting the clinical efficacy of them for chronic rhinosinusitis with nasal polyposis (CRSwNP).This positive effect could be attributed to their ability to target eosinophils, which drive the type 2 (T2) inflammation characteristic of CRSwNP. That was the main reason for being included in the present systematic review.

#### 4.4.6. Tezepelumab

A brief critical narrative review of data regarding existing research for biomarkers to assess tezepelumab’s efficacy in CRSwNP is presented in this paragraph.

Tezepelumab, an anti-Thymic Stromal Lymphopoietin (TSLP) biologic, is a quite recent add-on to biologic treatment for CRSwNP (FDA and EMA approval for CRSwNP: October 2025), so there was not adequate data to support the needs of the present systematic review in terms of the strict inclusion criteria initially established, by the time of final study selection. However, we decided to add this brief review for the specific agent mainly due to its increasing popularity among ENT doctors and existing supporting literature concerning potential biomarkers.

Potential predictive biomarkers for response to tezepelumab treatment:

• Peripheral Blood Eosinophils: A high baseline eosinophil count appears to be one of the most reliable predictors of a robust clinical response. Patients with type 2-skewed endotypes—often characterized by high eosinophils—typically show the strongest improvements in nasal polyp size and congestion.
• Baseline Plasma TSLP Levels: Clinical studies (such as trials for the anti-TSLP biologic CM326) indicate that patients with elevated baseline plasma TSLP levels (e.g., >330 fg/mL) achieve substantial reductions in nasal polyp scores [37].
• Fractional Exhaled Nitric Oxide (FeNO): Elevated FeNO levels are probably a strong marker for type 2 airway inflammation. Patients with higher FeNO often experience excellent symptom relief across both upper and lower airways [37,38,39].
• Total Serum IgE: High levels of Immunoglobulin E in the blood typically suggest active type 2 inflammation, making it a supportive biomarker for tezepelumab usage [37].
• Overall, biomarkers indicating high eosinophilic burden in nasal lining fluid and tissue appear to serve as good predictors of restored epithelial health and a reduction in the polyp size following tezepelumab treatment.

### 4.5. Local Allergic Rhinitis in the Era of Biologics

The immunopathological characteristics of local allergic rhinitis (LAR), including localized type 2 inflammation, mast cell and eosinophil activation, and the local production of allergen-specific IgE despite the absence of systemic atopy, suggest that this disease shares key inflammatory pathways with classical allergic rhinitis. Although there are no reported clinical studies evaluating biologic therapies in LAR, the described mechanisms provide a theoretical rationale for considering biologics targeting IgE or type 2 inflammatory cytokines in carefully selected patients with severe or refractory disease. Such an approach would require the accurate identification of individuals with active local allergic inflammation, highlighting the importance of reliable predictive biomarkers. A recent interesting scoping review [40] identifies several candidate markers, including local allergen-specific IgE detected in nasal secretions or lavage fluid, positive nasal allergen challenge and basophil activation tests, nasal eosinophilia, and increased local inflammatory mediators such as eosinophil cationic protein and tryptase, all of which reflect the underlying type 2 immune response. While these biomarkers are currently used primarily for diagnosis, they may also contribute to future patient stratification and selection for targeted biologic therapies once their predictive value for the treatment response has been established. Therefore, the development and validation of biomarker-driven approaches represent an important research priority that could facilitate personalized therapeutic strategies for LAR beyond conventional pharmacotherapy and allergen immunotherapy.

### 4.6. Controversial and Challenging Issues/Limitations of This Systematic Review

One of the most important limitations in finding well-documented predictive biomarkers is the fact that almost all original Phase-3 studies were focused on the efficacy and safety profile of the assessed agents rather than on investigating the role of possible predictive/prognostic biomarkers. This particular characteristic of all Phase-3 studies, along with the heterogeneity of available data (many different biomarkers, different specimens assessed, different biomarkers for the same biologic agents, different follow-up times for each agent/marker), is probably the main reason for the lack of meta-analyses, which would support evidence based recommendations towards the customized selection of biomarkers depending on the individual case.

An existing literature search clearly shows that there are no independent meta-analyses or large real-world studies focusing exclusively on using baseline biomarkers to select a specific biologic agent for CRSwNP.

In our point of view, the lack of other biomarker-exclusive selection studies is driven by two specific bottlenecks in current medical research:

1. The “Type 2” Blanket Problem

Every approved biologic for CRSwNP targets the exact same broad inflammatory profile: type 2 (T2) inflammation. Real-world studies use biomarkers like the blood eosinophil count (BEC) or total IgE exclusively as a yes/no switch to prove that a patient has T2 inflammation. Once the biomarkers confirm T2 inflammation, the data shows that the patient will likely respond to any of the biologics. The markers cannot definitively differentiate between them.

2. The Complete Absence of Head-to-Head Biomarker Trials

To publish a real-world study or meta-analysis showing which biomarker selects Agent A over Agent B, researchers need data where patients with specific biomarker levels were randomized to different drugs. No such head-to-head clinical trials exist. All available data comes from Indirect Network Meta-Analyses (NMAs). These statistical models only compare separate drug trials against each other.

On the other hand, post-hoc and secondary analyses of Phase-3 clinical trials theoretically could provide the strongest statistical evidence supporting biomarker-driven precision medicine in CRSwNP. However, their ability to guide the selection of a specific drug is still limited: although, they provide powerful evidence for within-drug prediction (identifying who responds best to Drug A), they struggle with between-drug selection (proving Drug A is better than Drug B for a specific patient).

While these analyses prove that biomarkers predict success, they cannot reliably tell a clinician “Based on these markers, prescribe Dupilumab instead of Mepolizumab.” The main reasons for this appear to be as follows:

1. The Lack of Head-to-Head Comparator Arms

All Phase-3 trials evaluated one active drug versus a placebo (e.g., dupilumab vs. placebo). No Phase-3 trial randomized patients to dupilumab vs. omalizumab vs. mepolizumab. Therefore, a secondary analysis can only prove that a biomarker predicts a response to that specific drug, but it cannot prove superiority over a competitor.

2. Overlapping Biomarker Profiles (The T2 Confound)

The primary biomarkers tracked in all Phase-3 trials (BEC, IgE, FeNO) are highly correlated with one another because they are all driven by the same upstream type 2 helper T-cell (Th2) pathway. A patient with severe CRSwNP will frequently have high BEC, high IgE, and high FeNO simultaneously. Because all three markers are elevated, secondary analyses show that this patient is an ideal candidate for all the biologics. The data does not provide a mathematical baseline to isolate one agent as the superior choice.

3. The “Prognostic” vs. “Predictive” Flaw

Many biomarkers identified in secondary analyses are actually prognostic (predicting how severe the disease is regardless of treatment) rather than predictive (predicting a specific response to a specific drug). High baseline eosinophils mean that a patient has highly aggressive, recurrent disease. They will show a massive drop in the polyp size based on a biologic simply because they had more polyps to lose, making it difficult to isolate true drug-specific efficacy.

Moreover, most biomarkers assessed in the current literature are generally reflecting the pathophysiology of the disease. However, they do not necessarily have any impact on the efficacy and durability of the biologic agent involved. That is probably the reason why the efficacy and safety of biologics in CRSwNP treatment are adequately documented but we are still quite far from adopting a number of biomarkers that we can rely on to justify the choice of the “ideal for the case” biologic. Consequently, at the moment, efficacy is the most used criterion to select the biologic drug for the treatment of CRSwNP disease, without further individualization.

The variety of mechanisms and molecular targets of monoclonal antibodies makes the previous issue even more complicated. Targeted and methodologically well-organized RCTs, establishing suitable primary endpoints exclusively related to specific biomarkers, are probably needed to completely investigate the role of possible biomarkers in predicting both good and poor responders to treatment.

Adequate follow-up time is also of paramount importance in order to document a correlation between a specific biomarker and the final result of the treatment of CRSwNP with any biologic agent. Permanent or temporary increases/decreases and varying behaviors of potential biomarkers through the time of the therapeutic management should be thoroughly examined and analyzed.

Many possible biomarkers in peripheral blood, tissue homogenates, nasal secretions, or even urine metabolites might be assessed. Therefore, it is considered extremely difficult to organize an RCT or even perform a conclusive systematic review or meta-analysis of such complicated data from several heterogeneous sources.

Despite the fact that several biomarkers seem to have documented prognostic/predictive value for the efficacy and durability of biologics in type 2 CRS treatment in general, there are no exact cut-off values of specific biomarkers for predicting the overall treatment response.

Given the heterogeneity of existing data and the lack of real-life studies for all potential biomarkers assessed, we suggest that the selection of such potential biomarkers and the most suitable biologic agent might be based upon the mechanism of action of every specific biologic agent in the inflammatory cascade, the profile (endotype) of the patient, and existing comorbidities. Each selection must be customized for each individual case in order to lead to a thorough precision medical treatment strategy.

## 5. Conclusions

Although biologic therapies have transformed the management of chronic rhinosinusitis with nasal polyps (CRSwNP), the development of clinically useful predictive biomarkers has progressed considerably slower than therapeutic innovation. The principal reason appears to be methodological rather than biological. Most pivotal Phase-3 randomized controlled trials were designed to establish efficacy and safety, with biomarker analyses incorporated only as exploratory or secondary endpoints. Consequently, available evidence predominantly identifies biomarkers associated with type 2 inflammation or disease severity rather than biomarkers capable of distinguishing which biologic is optimal for an individual patient.

Furthermore, the absence of head-to-head comparative trials, the substantial overlap between type 2 inflammatory biomarkers, the heterogeneity of biomarker assessment across studies, and the lack of standardized cut-off values have collectively limited the translation of biomarker research into clinical decision-making. Current biomarkers are more effective at identifying patients likely to benefit from biologic therapy in general than at guiding the selection of a specific biologic agent, highlighting the persistent gap between biomarker discovery and true precision medicine.

Future studies should therefore move beyond conventional efficacy-driven trial designs and adopt standardized biomarker-focused methodologies. Prospective studies should include predefined biomarker-related primary endpoints, longitudinal assessments of biomarker kinetics, standardized sampling protocols, and the integration of tissue, blood, and molecular profiling with clinical outcomes. Equally important will be comparative effectiveness studies and large multicenter registries capable of evaluating biomarker–treatment interactions under real-world conditions.

Looking ahead, an endotype-driven approach integrating molecular signatures, inflammatory pathways, transcriptomic and proteomic profiling, and relevant clinical phenotypes may provide a more robust framework for personalized treatment than reliance on isolated biomarkers alone. Such multidimensional strategies have the potential to redefine biologic selection in CRSwNP, shifting clinical practice from empirical drug choice toward mechanism-based precision medicine. Until such evidence becomes available, biologic selection should continue to be individualized based on disease characteristics, comorbidities, the mechanism of action, and shared clinical decision-making rather than on currently available biomarkers alone.

## Figures and Tables

**Table 1:** Potential biomarkers for all biologics assessed in this analysis.

Biologic Agent	Potential Biomarkers Assessed
**Omalizumab**	serum total IgE levels, eosinophils in nasal lavage
**Mepolizumab**	blood eosinophil counts, serum IL-5Ra, nasal IL-5Ra and serum ECP
**Dupilumab**	blood eosinophil count, serum total IgE, TARC, PARC, plasma periostin, plasma eotaxin-3, eosinophil cationic protein (ECP) concentrations, total IgE in nasal secretions, serum PGD_2_, 24 h urinary leukotriene E4 (LTE4)
**Benralizumab**	blood eosinophils and basophil counts
**Reslizumab**	peripheral blood eosinophil counts, peripheral blood and nasal secretions of IL-3, IL-5, SOL IL-1Ra, Eotaxins 1,2,3, ECP, GM-CSF

ECP: eosinophil cationic protein, TARC: thymus and activation-regulated chemokine/also seen as CCL17, PARC: pulmonary and activation-regulated chemokine, GM-CSF: granulocyte macrophage-colony stimulating factor, PGD: phosphogluconate dehydrogenase, LTE4: leukotriene E4.

**Table 2:** All existing original “MOTHER” studies (RCT’S) assessing the efficacy and safety of omalizumab, mepolizumab, dupilumab, benralizumab, and reslizumab in CRSwNP treatment (studies performing pool data analyses or responder analysis using data from “mother studies” are not included in this table).

Original Rct	Biologic agent Tested in Crswnp	Target	Inclusion/Eligibility Criteria/Endpoints	Outcomes in General
**SINUS-24 & SINUS 52**	Dupilumab	Il-4 receptor alpha (Ra), Il-13	CRSwNP, NPS > 5, nasal polyposis refractory to standard treatment (systemic steroids or/and INCS) in last two years or prior sinonasal surgery	Statistically significant improvement on NPS, L-K score, SNOT-22, UPSIT vs. placebo
**POLYP 1 & POLYP 2**	Omalizumab	IgE	Nasal polyposis refractory to 4 weeks INCS treatment, NPS ≥ 5, NCS > 2, SNOT-22 ≥ 20	Statistically significant improvement on NCS, SNOT-22, UPSIT and total nasal symptom score, reduced need for rescue surgery at week 24 vs. placebo
**SYNAPSE**	Mepolizumab	Il-5	CRSwNP, NPS > 5, nasal polyposis refractory to standard treatment (systemic steroids or/and INCS), VAS nasal obstruction score > 5, at least one prior sinonasal surgery	Statistically significant improvement on NPS, NCS, SNOT 22, decreased need for rescue surgery and blood eosinophil count vs. placebo
**OSTRO**	Benralizumab	Il-5Ra	Severe CRSwNP, who were symptomatic despite treatment with intranasal corticosteroids and who had a history of systemic corticosteroid (SCS) use and/or surgery for nasal polyps.	Significant improvement in difficulty in sense of smell score at week 40. Subgroup analyses suggested influences of co-morbid asthma, number of NP surgeries, sex and body mass index
**Gevaert et al. 2006** [5]	Reslizumab	Il-5	Bilateral nasal polyposis grade 3–4 or recurrent nasal polyps after surgery	Individual nasal polyp scores improved only in half of the treated patients for 4 weeks.

**Table 3:** Evidence for possible biomarkers to predict the efficacy of biologic agents in CRSwNP: available data from existing original biggest trials in the field (“MOTHER” RCT’s).

Biologic Agent Tested in Crswnp	“Mother” Rct’(s)	Authors/Year	Possible Biomarkers Assessed	Conclusive Results About Biomarkers in Brief
**Omalizumab**	POLYP-1 (NCT03280550) POLYP-2 (NCT03280537)	Gevaert et al. 2020 [8]	Serum IgE levels were determined at baseline	No specific results reported in the original RCTs.
**Mepolizumab**	SYNAPSE (NCT03085797)	Bachert et al. 2022 [10]	Baseline blood eosinophil count	No specific results reported in the original RCT.
**Dupilumab**	SINUS-24 (NCT02912468) SINUS-52 (NCT02898454)	Bachert et al. 2019 [7]	(Parameters assessed For SINUS-52): blood eosinophil count, serum total IgE, thymus and activation regulated chemokine (TARC), periostin, plasma eotaxin-3 concentrations, eosinophil cationic protein (ECP), and eotaxin-3 concentrations and total IgE in nasal secretions were also assessed	No specific results reported in the original RCTs.
**Benralizumab**	OSTRO (NCT:03401229)	Emson et al. 2024 [15]	Blood eosinophil and basophil counts	Subgroup analyses suggested influences baseline blood eosinophil count on treatment effects.
**Reslizumab**	Randomized Controlled Trial	Gevaert et al. 2006 [5]	Peripheral blood eosinophil counts and peripheral blood and nasal secretions of IL-3, IL-5, SOL IL-RRa, eotaxin, ECP, and GM-CSF	Blood eosinophil numbers and concentrations of eosinophil cationic protein were reduced up to 8 weeks after treatment in serum and nasal secretions. Responders had increased IL-5 concentrations in nasal secretions at baseline compared with non-responders. Logistic regression analysis revealed that increased nasal IL-5 levels (>40 pg/mL) predict the response to anti-IL-5 treatment.

**Table 4:** Biologic agents assessed in the present review: status of FDA *’s and European Commission (EC) *’s approval for use in CRSwNP.

Biologic Agent/Brand Name	Status of FDA/European Commission’s (Ema) Approval for Crswnp	Date of Approval	Target
Omalizumab/XOLAIR^®^	APPROVED by FDA * and EC (EMA) *	1 December 2020/6 August 2020	IgE
Dupilumab/DUPIXENT^®^	APPROVED by FDA and EC (EMA)	26 June 2019/29 October 2019	IL-4 Receptor Alpha (Ra)
Mepolizumab/NUCALA^®^	APPROVED by FDA and EC (EMA)	29 July 2021/17 November 2021	IL-5
Reslizumab/CINQAIR/CINQAERO^®^	NOT APPROVED (IN PHASE-3 TRIALS), FDA/EC (EMA) APPROVED FOR SEVERE ASTHMA	-	IL-5
Benralizumab/FASENRA^®^	NOT APPROVED (IN PHASE-2 TRIALS), FDA/EC (EMA) APPROVED FOR SEVERE ASTHMA	-	IL-5Ra

* FDA: Federal Drug Association, EC: European Community.

## Data Availability

Data is contained within the article or Supplementary Material. The original contributions presented in this study are included in the article/Supplementary Material. Further inquiries can be directed to the corresponding author.

## Supplementary Materials

The following supporting information can be downloaded at: https://www.mdpi.com/article/10.3390/medicina62061188/s1, PRISMA 2020 Checklist; Table S1: All (12) RCT’s assessing efficacy and safety of omalizumab, dupilumab, mepolizumab, benralizumab and reslizumab in the treatment of CRSwNP included in the present systematic review. All those studies use at least one possible biomarker (defined as a primary or secondary endpoint) as outcome’s prognostic factor and/or efficacy indicator for the specific biologic agent tested (such biomarkers and relevant data are marked in yellow colour); Table S2: All (13) systematic reviews/reviews/indirect treatment comparison studies assessing efficacy and safety of omalizumab, dupilumab, mepolizumab, benralizumab and reslizumab in the treatment of CRSwNP, included in the present systematic review. All those studies summarize and discuss the existing data regarding several possible biomarkers as outcome’s prognostic factors and/or efficacy indicators for the specific biologic agent assessed (biomarkers/relevant data are marked in yellow colour).
