# Supplementary material for: The Use of Biomarkers to Justify the Choice of the Proper Biologic Agent for the Treatment of Chronic Rhinosinusitis with Nasal Polyps: A Systematic Review
Source: Medicina (Kaunas). 2026 Jun 18;62(6):1188. doi: 10.3390/medicina62061188 (PMC13304456; doi:10.3390/medicina62061188)
Supplement: Supplementary file 1 [file medicina-62-01188-s001.zip › medicina-4339049-Table S1.pdf]

Table S1: **All (12) RCT's assessing efficacy and safety of omalizumab, dupilumab, mepolizumab, benralizumab and reslizumab in the treatment of CRSwNP included in the present systematic review. All those studies use at least one possible biomarker (defined as a primary or secondary endpoint) as outcome's prognostic factor and/or efficacy indicator for the specific biologic agent tested (such biomarkers and relevant data are marked in yellow colour).**

CRSwNP: Chronic rhinosinusitis with nasal polyps, NPS: Nasal Polyp Score, NCS: Nasal Congestion Score, SNOT-20,22: Sinonasal Outcome Test Questionnaire, VAS: Visual Analogue Symptom Scale, UPSIT: the University of Pennsylvania Smell Identification Test, LoS: Loss of smell patient-reported test, DSS: Difficulties in sense of smell, L-M CT score: Lund Mackay imaging (Computed Tomography-based) score, PNIF: Peak Nasal Inspiratory Flow, FEV_1: Forced Expiratory Volume in one second, FVC: Forced Vital Capacity, PEFR: Peak Expiratory Flow Rate, AQLQ: Asthma Quality of Life Questionnaire, ACQ-6: Asthma Control Questionnaire, TNSS: Total Nasal Symptoms Score Questionnaire, IgE: Immmunoglobulin E. ROCS: rescue oral corticosteroids, SCS: Systemic corticosteroids, NFS: Need for rescue surgery (endoscopic sinus surgery), IL-(1b,2,3,4,5,6,10,13,17): Interleukin (1b,2,3,4,5,6,10,13,17), CCL11, CCL24 and CCL26**:** Eotaxins 1,2,3, ECP: Eosinophil cationic protein, TNF-a: Tumor necrosis factor-a, TARC: Thymus and activation-regulated chemokine, PARC: Pulmonary and activation-regulated chemokine, GM-CSF: Granulocyte macrophage-colony stimulating factor, PGD: Phosphogluconate dehydrogenase, LTE4:Leukotriene E4.

Author/researcher/ year of publication	Aim of study<br>End points/Parameters assessed	Type of study<br>n (patients)	Name/ID of Study/Regimen given/tested	Follow up time	Outcomes in brief
Gevaert et al 2006 [5]	Phase I, single-dose, randomized, double-blind, placebo-controlled, 3-arm, parallel-group, 2-center safety and pharmacokinetic study of reslizumab in patients with nasal polyps.<br><br><u>Outcome measures:</u><br><br>Total Nasal Polyp Score, NPIF measurements and symptoms (anterior rhinorhea, nasal obstruction, postnasal drip, loss of smell) were recorded.<br><br>Peripheral blood eosinophil counts and peripheral bloos and nasal secretions of IL-3, IL-5, SOL IL-RRa, Eotaxin, ECP, GM-CSF	Phase I, single-dose, randomized, double-blind, placebo-controlled, 3-arm, parallel-group, 2-center safety and pharmacokinetic study of reslizumab in patients with nasal polyps.<br><br>**-Reslizumab vs placebo (1 study) n=24 (8 patients per arm)**	**-Reslizumab vs placebo (1 study) n=24**<br><br>After a 1- to 2-week run-in period, subjects were randomized to receive treatment with reslizumab at 3 mg/kg or 1 mg/kg or placebo.<br><br>A single dose was administered as an intravenous infusion over 30 minutes in a double-blind fashion.<br><br>Subjects were confined to the study site for 24 hours after dosing for safety evaluations and collection of samples for pharmacokinetic analyses.	Follow-up visits were scheduled 48 hours after dosing and 1, 2, 4, 8, 12, 16, 20, 24, 28, 32, and 36 weeks after dosing.	Blood eosinophil numbers and concentrations of eosinophil cationic protein were reduced up to 8 weeks after treatment in serum and nasal secretions.<br><br>Individual nasal polyp scores improved only in half of the treated patients for 4 weeks.<br><br>Responders had **increased IL-5 concentrations in nasal secretions at baseline compared with non-responders**.<br>Logistic regression analysis revealed **that increased nasal IL-5 levels (>40 pg/mL) predict the response to anti-IL-5 treatment.**

Author/researcher/ year of publication	Aim of study<br>End points/Parameters assessed	Type of study<br>n (patients)	Name/ID of Study/Regimen given/tested	Follow up time	Outcomes in brief
Jonstam Karin et al<br><br>2019 [6]	Report the local effect of dupilumab on type-2 inflammatory biomarkers in nasal secretions and nasal polyp tissues of patients with CRSwNP<br><br>Eotaxin-3, total-IgE and ECP in nasal secretions,<br>ECP, total IgE, IL-4, IL-5, IL-17, TNF-a, IL-10, IL-1b, IL-6, IL-13, TARC, eotaxin-3, eotaxin-2, eotaxin-1, PARC in tissue homogenates<br><br>Primary efficacy endpoint was mean change in bilateral endoscopic NPS from baseline to week 16.<br>Secondary endpoints: Lund-Mckay CT score, % of maxillary sinus volume occupied by disease, SNOT-22, UPSIT, PNIF, patient-rated nasal congestion, anterior/posterior rhinorrhea, loss of smell, nocturnal awakenings and overall symptom severity (VAS).<br>Safety outcomes were also assessed.<br>Most common treatment –emergent adverse events were nasopharyngitis, injection-site reaction and headache	Randomized-double blind, placebo-controlled, parallel group study<br><br>n=60<br><br>Dupilumab<br>n=30<br><br>Placebo<br>n=30	(NCT01920893)<br><br>After 4 weeks of treatment with intranasal mometasone furoate,<br>60 patients were randomly allocated to receive either<br>600mg loading dose of dupilumab followed by 300mg dupilumab (n=30)<br>or placebo (n=30), weekly, for 16 weeks	16 weeks	Dupilumab treatment reduced multiple biomarkers of type-2 inflammation in nasal secretions and polyp tissues in patients with CRSwNP.<br><br>Antagonism of IL-4Ra signaling suppresses IL-4/IL-13 dependent processes, such as mucosal IgE formation and the expression of chemokines

Bachert Claus et al<br><br>2019 [7]	Efficacy and safety of dupilumab in patients with severe chronic rhinosinusitis with nasal polyps<br><br>The coprimary endpoints in both studies were: change from baseline in both endoscopic NPS and nasal congestion severity at week 24<br><br>Key secondary endpoints were: change from baseline at week 24 in sinus opacification assessed by Lund Mackay CT score, patients reported total symptom score, loss of smell, daily loss of smell or smell impairment, SNOT-22 score and UPSIT, Pooled data from the two studies was used to assess the proportion of patients requiring rescue SCS or surgery<br><br>Extra parameters assessed For SINUS-52: blood eosinophil count, serum total IgE, thymus and activation regulated chemokine (TARC), periostin, plasma eotaxin-3 concentrations, eosinophil cationic protein (ECP) and eotaxin-3 concentrations and total IgE in nasal secretions were also assessed<br>Safety- adverse events were assessed for both studies until week 24	Data analysis from two multicentre, randomized, double-blind, placebo-controlled, parallel-group phase 3 trials:<br><br>LIBERTY NP SINUS-24, n=276<br>&<br>LIBERTY NP SINUS-52, n=448	LIBERTY NP SINUS-24 (NCT02912468):<br>276 patients (on a background therapy of mometasone furoate nasal spray);<br>133 assigned to placebo every 2 weeks and 143 assigned to dupilumab 300mg every 2 weeks for 24 weeks<br><br>LIBERTY NP SINUS-52 (NCT02898454) :<br>448 patients (on a background therapy of mometasone furoate nasal spray);<br><br>153 assigned to placebo every 2 weeks for 24 weeks<br><br>145 assigned to dupilumab 300mg every 2 weeks until week 24 and every 4 weeks from week 24 to 52<br><br>150 assigned to dupilumab 300mg every 2 weeks for 52 weeks	24 weeks<br><br>52 weeks	Dupilumab significantly improved the coprimary endpoints in both studies.<br><br>Dupilumab reduced polyp size, sinuses opacification and severity of symptoms<br><br>Regimens were well tolerated in both studies<br><br>The most common adverse events were (nasophanryngitis, worsening of nasal polyps and asthma, headache, epistaxis and injection-site erythema) but were more frequent in placebo group
Gevaert P et al<br><br>2020 [8]	Efficacy and safety of omalizumab in patients with inadequately controlled CRSwNP despite daily INCS therapy<br><br>Coprimary endpoints in both studies were : change from baseline to week 24 on Nasal Polyp Score (NPS) and Nasal Congestion Score (NCS)<br><br>Secondary endpoints were: change from baseline to week 24 in SNOT-22 score, UPSIT, sense of smell, postnasal drip, runny nose, rescue SCS or surgery and adverse events<br><br>Parameters also assessed: Asthma Quality of Life Questionnaire (AQLQ), Health-related quality of life (HRQoL), Total Nasal Symptom Score (TNSS).<br>Blood samples were collected at screening and at weeks 16, 24 and 28 for routine analysis, serum IgE levels were determined at baseline.	Data/Results analysis from two replicate, multicentre, randomized, double-blind, placebo-controlled, phase 3 studies<br><br>POLYP-1<br>n=138<br><br>&<br><br>POLYP-2<br>n=127	POLYP-1 (NCT03280550): 138 patients**;**<br>66 assigned to placebo every 2-4 weeks and backround intranasal mometasone for 24 weeks<br><br>72 assigned to omalizumab (75 to 600mg every 2-4 weeks depending on pretreatment total IgE and body weight) and background intranasal mometasone for 24 weeks<br><br>POLYP-2 (NCT03280537): 127 patients;<br>65 assigned to placebo every 2-4 weeks and backround intranasal mometasone for 24 weeks<br><br>62 assigned to omalizumab (75 to 600mg every 2-4 weeks depending on pretreatment total IgE and body weight) and background intranasal mometasone for 24 weeks	24 weeks<br><br>additional 28 weeks in open label extension study	POLYP-1 & POLYP-2 met both coprimary endpoints and demonstrated significant improvement in NPS and mean daily NCS as well as patient-reported assessments of severity of symptoms in response to omalizumab vs placebo on a background of intranasal mometasone at week 24<br><br>Omalizumab was well tolerated and AE's were consistent with those preciously reported

Author/researcher/ year of publication	Aim of study End points/Parameters assessed	Type of study n (patients)	Name/ID of study/Regimen given/tested	Follow up time	Outcomes in brief
Gevaert Phillip et al<br><br>2022 [9]	To assess long-term efficacy safety and durability of omalizumab for nasal polyposis in an open-label extension study, in adults with CRSwNP who completed POLYP1 or 2<br><br>Co-primary endpoints: change from baseline to week 24 on Nasal Polyp Score (NPS) and Nasal Congestion Score (NCS)<br><br>Secondary outcomes: patient-recorded efficacy outcomes of nasal congestion, sense of smell, postnasal drip, runny nose:<br>TNSS SCORE, NCS, SNOT-22, UPSIT, AQLQ<br><br>Adverse effects and concomitant medication were also monitored throughout the OLE<br><br>Exploratory endpoints: rescue SCS, need for rescue surgery<br><br>Blood samples were collected at screening and at weeks 16, 24 and 28 for routine analysis, serum IgE levels were determined at baseline.	Open-label extension study<br>Patients and data from two replicate, multicentre, randomized, double-blind, placebo-controlled, phase 3 studies<br>POLYP-1, n=138 & POLYP-2, n=127<br><br>Open label Extension study<br>n=249	POLYP-1 (NCT03280550): 138 patients**;**<br>66 assigned to placebo every 2-4 weeks and backround intranasal mometasone for 24 weeks<br>72 assigned to omalizumab (75 to 600mg every 2-4 weeks depending on pretreatment total IgE and body weight) and background intranasal mometasone for 24 weeks<br>POLYP-2 (NCT03280557): 127 patients;<br>65 assigned to placebo every 2-4 weeks and backround intranasal mometasone for 24 weeks<br>62 assigned to omalizumab (75 to 600mg every 2-4 weeks depending on pretreatment total IgE and body weight) and background intranasal mometasone for 24 weeks<br><br>**-OLE protocol**: Patients initially randomized to omalizumab (POLYP1 AND 2) continued to receive omalizumab for 28 additional weeks (from weeks 24 to 52), for 52 total weeks of treatment and are referred to as patients who continued omalizumab. Patients initially randomized to placebo in POLYP 1 AND 2, received omalizumab for 28 weeks (from weeks 24 to 52) and are referred to a patients who switched treatment<br>OLE Protocol-specified study dosing was 75 to 600mg every 2-4 weeks, based on serum total IgE and body weight	Omalizumab was withdrawn after the treatment period and patients were observed for an additional 24 weeks (from weeks 52 to 76)<br><br>All patients continued to receive background mometasone furoate	The efficacy and safety profile from this study supports extended omalizumab treatment up to 1 year for CRSwNP with inadequate response to nasal corticosteroids

Author/researcher/ year of publication	Aim of study<br>End points/Parameters assessed	Type of study<br>n (patients)	Name/ID of study/Regimen given/tested	Follow up time	Outcomes in brief
Bachert Claus et al<br><br>2022 [10]	Mepolizumab for CRSwNP: Treatment efficacy by co-morbidity and blood eosinophil count<br><br>Subgroup analysis to assess the efficacy of mepolizumab in patients from SYNAPSE study, grouped by co-morbid asthma, aspirin-exacerbated respiratory disease (AERD), and baseline blood eosinophil count (BEC)<br><br>Co-primary endpoints:<br>-change from baseline in total endoscopic NP score at week 52 and change from baseline in total obstruction VAS score during weeks 49-52<br><br>Secondary endpoints:<br>-time-to-first actual nasal surgery up to week 52<br>-proportion of patients requiring SCS for NP up to week 52<br>-change from baseline in overall symptoms VAS score during weeks 49-52<br>-SNOT-22 score at week 52<br>-composite VAS score during weeks 49-52<br>-VAS score for loss of smell during weeks 49-52<br>-AQLQ score at week 52 (in asthma patients)<br><br>Safety assessment by monitoring for AE's	Data analysis from a randomized double-blind, placebo-controlled, phase-3, multicentre study<br><br>n= 407	SYNAPSE (NCT03085797):<br><br>Mepolizumab 100mg added to standard of care compared to placebo added to standard of care, every 4 weeks for 52 weeks<br><br>414 patients were randomly assigned with 407 included in the ITT (intention to treat) population;<br><br>206 received mepolizumab<br><br>201 received placebo	52 weeks	Mepolizumab reduced polyp size and nasal obstruction in CRSwNP regardless of the presence of co-morbid asthma or AERD

Author/researcher/ year of publication	Aim of study<br>End points/Parameters assessed	Type of study<br>n (patients)	Name/ID of Study/Regimen given/tested	Follow up time	Outcomes in brief
Bachert C. et al. 2017 [11]	Reduced need for surgery in severe nasal polyposis with mepolizumab, randomized trial.<br><br>The primary endpoint was the number of patients no longer requiring surgery at Week 25, based on a composite endpoint of endoscopic nasal polyp score and nasal polyposis severity visual analogue scale (VAS) score.<br>Secondary endpoints: change in nasal polyposis severity VAS score, endoscopic nasal polyp score, improvement in individual VAS symptoms (rhinorrhea, mucus in throat, nasal blockage, and sense of smell), patient-reported outcomes (SNOT-22) and safety.<br>The EuroQual 5-dimensions[EQ-5D] questionnaire), peak nasal inspiratory flow (PnIF), olfaction testing (performed using the Sniffin' Sticks Screening-12 test), lung function assessments (forced expiratory volume in 1 second [FEV_1], forced vital capacity [FVC], and peak expiratory flow rate [PEFR]), blood eosinophil counts, and pharmacokinetics.	Randomized, double-blind, placebo-controlled, multicentre study<br><br>n=105	NCT01362244<br><br>Patients received intravenous **mepolizumab** 750 mg or placebo every 4 weeks for a total of six doses, in addition to daily topical corticosteroid treatment.<br><br>105 patients received mepolizumab (n=54) or placebo (n=51)		A significantly greater proportion of patients in the mepolizumab group compared with the placebo group no longer required surgery at Week 25 (16[30%] vs 5[10%], respectively.<br><br>There was a significant improvement in nasal polyposis severity VAS score, endoscopic nasal polyp score, all individual VAS symptom scores, and sino-nasal outcome test (SNOT-22) score in the mepolizumab group, compared with placebo.<br><br>Mepolizumab's safety profile was comparable to placebo.
Fujieda Shigeharu et al<br><br>2022 [12]	To demonstrate that dupilumab efficacy in patients with CRSwNP from SINUS-52 study is unaffected by eosinophilic status<br><br>Co-primary efficacy endpoints: changes from baseline at week 24 in NPS, NCS and Lund Mackay CT SCORE (in Japan patients only)<br>Secondary endpoints: NPS, NCS from baseline at 52 weeks, changes from baseline at week 24 & 52 in SNOT-22, patient-reported Total Symptom Score (TSS), VAS score and UPSIT<br><br>A sub-analysis was conducted to determine the effect of dupilumab on blood eosinophil count in each Eosinophilic Chronic Rhinosinusitis Status (ECRS) subgroup	Data from a multicentre, randomized, double-blind, placebo-controlled, parallel-group phase 3 trials:<br><br>LIBERTY NP SINUS-52, n=448	LIBERTY NP SINUS-52 (NCT02898454) :<br>448 patients (on a background therapy of mometasone furoate nasal spray);<br>153 assigned to placebo every 2 weeks for 24 weeks<br>145 assigned to dupilumab 300mg every 2 weeks until week 24 and every 4 weeks from week 24 to 52<br>150 assigned to dupilumab 300mg every 2 weeks for 52 weeks	24 weeks<br><br>52 weeks	Dupilumab produced consistent improvement in symptoms of severe CRSwNP irrespective of ECRS status.<br><br>Blood eosinohil level may not be a suitable marker for dupilumab efficacy in CRSwNP

Author/researcher/ year of publication	Aim of study End points/Parameters assessed	Type of study n (patients)	Name/ID of Study/Regimen given/tested	Follow up time	Outcomes in brief
Hopkins Claire et al<br><br>2021 [13]	To assess the efficacy of dupilumab in patients with a history of prior sinus surgery for CRSwNP<br>The co-primary endpoints were: change from baseline in both endoscopic NPS and nasal congestion severity at week 24<br>Key secondary endpoints were: change from baseline at week 24 in sinus, opacification assessed by Lund Mackay CT score, patients reported total symptom score, loss of smell, daily loss of smell or smell impairment, SNOT-22 score, UPSIT, rescue SCS or surgery<br>Extra parameters assessed For SINUS-52: blood eosinophil count, serum total IgE, TARC, periostin, plasma eotaxin-3 concentrations, ECP and eotaxin-3 concentrations and total IgE in nasal secretions<br>Safety- adverse events were assessed for both studies up to week 24	Results/data analysis from two multicentre, randomized, double-blind, placebo-controlled, parallel-group phase 3 trials:<br>n=724<br>LIBERTY NP SINUS-24, n=276<br>&<br>LIBERTY NP SINUS-52, n=448	LIBERTY NP SINUS-24 (NCT02912468):<br>276 patients (on a background therapy of mometasone furoate nasal spray);<br>133 assigned to placebo every 2 weeks and 143 assigned to dupilumab 300mg every 2 weeks for 24 weeks<br>LIBERTY NP SINUS-52 (NCT02898454) :<br>448 patients (on a background therapy of mometasone furoate nasal spray);<br>153 assigned to placebo every 2 weeks for 24 weeks<br>145 assigned to dupilumab 300mg every 2 weeks until week 24 and every 4 weeks from week 24 to 52<br>150 assigned to dupilumab 300mg every 2 weeks for 52 weeks	24 weeks<br><br>52 weeks	Dupilumab improved CRSwNP outcomes irrespective of surgery history, with greater improvements in endoscopic outcomes in patients with shorter duration since last surgery
Mustafa Shahzad et al<br><br>2021 [14]	Dupilumab as Add-on Therapy for CRSwNP in Aspirin exacerbated Respiratory Disease<br><br>Primary outcomes: change in SNOT-22 from baseline to after six months of therapy<br><br>Secondary outcomes: change from baseline to completion of the study for Lund-Mackay scores, UPSIT, Asthma Control Test (ACT), AQLQ, and biomarkers (total serum IgE, serum tryptase, serum thymus and activation regulated cytokine, serum PGD_2 and 24-hour urinary leukotriene E_4 (LTE_4)	Prospective case-series with placebo run-in phase<br><br>n=10	All patients (n=10) received 4 weeks of placebo, followed by 6 months of Dupilumab 300mg/2ml every 14±3 days	6 months	Dupilumab was very effective as add-on therapy for CRSwNP in AERD,signigficantly improving patient-reported outcomes, sinus opacification and markers of T2 inflammation

Author/researcher/ year of publication	Aim of study End points/Parameters assessed	Type of study n (patients)	Name/ID of Study/Regimen given/tested	Follow up time	Outcomes in brief
Emson C. 2024 [15]	The phase-3 OSTRO study enrolled patients with severe CRSwN: bilateral NP and a total NPS of >5 (with unilateral scores of >2) despite maintenance treatment with INCS for at least 4 weeks before enrollment and a history of SCS use and/or surgery for NP.<br><br>In addition, patients were required to have ongoing NP symptoms for >12 weeks, moderate to severe nasal blockage (nasal blockage score [NBS] >2, captured electronically in the Nasal Polyposis Symptom Diary), and Sinonasal Outcome Test 22 (SNOT-22) total score >30 at enrollment.<br><br><u>End points</u>: NPS, SNOT-22, time to first NP surgery AND/OR SCS use for NP, time to first NP surgery, DSS score, L-M CT score, SCS use, UPSIT, ACQ-6 score, blood eosinophils and basophil counts<br><br>Safety assessments included treatment-emergent adverse events (AEs), laboratory variables, and antidrug antibody (ADA) assays.	Phase 3-OSTRO study<br><br>Benralizumab vs placebo<br><br>randomized, double-blind, placebo-controlled, parallel-group phase-3 trial	OSTRO study<br><u>(NCT: 03401229)</u><br><br>The study population comprised 413 randomized patients (207 in the benralizumab group and 206 in the placebo group).<br><br>Patients were randomized 1:1 to treatment with benralizumab 30 mg or placebo every 4 weeks for the first 3 doses and every 8 weeks thereafter.	40 weeks<br><br>56 weeks<br><br>Responder analysis for NPS at weeks: 24, 40 and 56	Benralizumab significantly improved NPS and nasal blockage score compared to placebo at week 40 ($P \leq .005$). Improvements in Sinonasal Outcome Test (SNOT-22) score at week 40, time to first NP surgery and/or SCS use for NP, and time to first NP surgery were not statistically significant between treatment groups<br><br>Nominal significance was obtained for improvement in difficulty in sense of smell score at week 40.<br>As expected, blood eosinophils were nearly completely depleted and basophil counts were reduced in the benralizumab group.<br><br>Subgroup analyses suggested influences of co morbid asthma, number of NP surgeries, sex, body mass index, and baseline blood eosinophil count on treatment effects.<br>Benralizumab was safe and well tolerated.

Author/researcher/ year of publication	Aim of study<br>End points/Parameters assessed	Type of study<br>n (patients)	Name/ID of Study/Regimen given/tested	Follow up time	Outcomes in brief
Pinto et al 2010 [16]	A randomized, double blind, placebo-controlled trial of anti-IgE treatment (omalizumab) vs placebo for chronic rhinosinusitis.<br><br>Patients included reported sinonasal symptoms for greater than 12 weeks and also had confirmatory findings on nasal endoscopy and evidence of inflammation on sinus CT scan. Serum total IgE between 30-700 IU/ml was also required.<br><br>The study was performed in patients with CRS despite treatment (including surgery).<br><br><u>Primary outcome measures:</u> sinus inflammation, as determined by CT imaging<br><br><u>Secondary outcome measures:</u><br>TNSS, SF-36, SNOT-20, UPSIT,NPIF, inflammation in nasal lavage (eosinophils).	Randomized, double blind, placebo-controlled trial<br><br>Omalizumab vs placebo	<u>NCT: 00117611</u><br><br>The study population finally comprised 14 randomized patients<br><br>n=7 in the omalizumab group and<br><br>n=7 in the placebo group	24 weeks	A significant reduction in inflammation was found for the omalizumab group from baseline to end of follow-up.<br><br>SNOT-20: median change in score was consistent with a clinical significant improvement in the omalizumab group.<br><br>UPSIT: Net change in total UPSIT score was not statistically different between the groups.<br><br>NPIF: : Net change in total score was not statistically different between the groups.<br><br>TNSS: No significant net difference across treatments.<br><br>Eosinophils in nasal lavage: there were no differences in net change between the treated groups.
