# Supplementary material for: The Use of Biomarkers to Justify the Choice of the Proper Biologic Agent for the Treatment of Chronic Rhinosinusitis with Nasal Polyps: A Systematic Review
Source: Medicina (Kaunas). 2026 Jun 18;62(6):1188. doi: 10.3390/medicina62061188 (PMC13304456; doi:10.3390/medicina62061188)
Supplement: Supplementary file 1 [file medicina-62-01188-s001.zip › medicina-4339049-Table S2.pdf]

Table S2: All (13) systematic reviews/reviews/indirect treatment comparison studies assessing efficacy and safety of omalizumab, dupilumab, mepolizumab, benralizumab and reslizumab in the treatment of CRSwNP, included in the present systematic review. All those studies summarize and discuss the existing data regarding several possible biomarkers as outcome's prognostic factors and/or efficacy indicators for the specific biologic agent assessed (biomarkers/relevant data are marked in yellow colour).

CRSwNP: Chronic rhinosinusitis with nasal polyps, NPS: Nasal Polyp Score, NCS: Nasal Congestion Score, SNOT-20,22: Sinonasal Outcome Test Questionnaire, VAS: Visual Analogue Symptom Scale, UPSIT: the University of Pennsylvania Smell Identification Test, LoS: Loss of smell patient-reported test, DSS: Difficulties in sense of smell, L-M CT score: Lund Mackay imaging (Computed Tomography-based) score, PNIF: Peak Nasal Inspiratory Flow, FEV_1: Forced Expiratory Volume in one second, FVC: Forced Vital Capacity, PEFR: Peak Expiratory Flow Rate, AQLQ: Asthma Quality of Life Questionnaire, ACQ-6: Asthma Control Questionnaire, SF-36: The 36-Item Short Form Health Survey, RSOM-31: Rhinosinusitis Outcome Measure, EQ-5D: Euro-quality-of-life descriptive system, TNSS: Total Nasal Symptoms Score Questionnaire, IgE: Immmunoglobulin E. ROCS: rescue oral corticosteroids, SCS: Systemic corticosteroids, NFS: Need for rescue surgery (endoscopic sinus surgery), IL-(1b,2,3,4,5,6,10,13,17,33): Interleukin (1b,2,3,4,5,6,10,13,17), CCL11, CCL24 and CCL26**:** Eotaxins 1,2,3, ECP: Eosinophil cationic protein, TNF-a: Tumor necrosis factor-a, TARC: Thymus and activation-regulated chemokine/also seen as CCL17, PARC: Pulmonary and activation-regulated chemokine, GM-CSF: Granulocyte macrophage-colony stimulating factor, PGD: Phosphogluconate dehydrogenase, LTE4:Leukotriene E4, MIP 1b: macrophage inflammatory protein 1b, MFNS: mometasone furoate nasal spray, N-ERD: non steroidal anti-inflammatory drugs-exacerbated respiratory disease.

Authors/researchers/ year of publication	Aim of study/parameters assessed	Type of study	Trials and patients included	Outcomes in brief
Tsetsos N et al [17]<br><br>2018	Monoclonal antibodies for the treatment of chronic rhinosinusuitis with nasal polyps (CRSwNP)<br>Parameters assessed:<br>Total Nasal Endoscopic Polyp Score, CT score, Quality of life measures (SNOT-20, SNOT-22, SF-36, Rhinosinusitis Outcome Measurement Instrument (RSOM-31)<br><br>Nasal Airflow (PNIF), Olfaction (UPSIT)<br><br>Type 2 helper T-cell-associated biomarkers<br><br>Safety and adverse events	Systematic review	6 RCT's n=257<br><br>-Omalizumab (2 studies) n= 24, 14<br><br>-Reslizumab (1 study) n=24<br><br>-Mepolizumab (2 studies) n=105, 30<br><br>-Dupilumab (1 study) n=60	Biologic agents proved to be effective in reducing TPS (total nasal endoscopic polyp score). They also brought improvement in opacification on CT, quality of life measures, nasal airflow<br>and olfaction.<br>All agents were well tolerated.<br><br>Concerning changes in Type 2 Helper T-cell-associated biomarkers, despite the significant decrease of eosinophil counts which was reported in both treatment groups with the use of reslizumab, a rebound eosinophilia was appeared at week 24 and week 32 in 1mg/kg and 3mg/kg treatment groups, respectively.<br>The use of mepolizumab lead to a significant reduction in blood eosinophil counts compared to placebo group and in this case no rebound eosinophilia effect was reported in any of the two RCTs.<br><br>No statistically significant decrease in eosinophil counts was also reported for both dupilumab and placebo group in Bachert et al. study (p=0,78).<br>Finally, in Pinto et al. study, omalizumab did not show a significant difference in eosinophils in nasal lavage in either group (p<0,47).<br><br>Some others biomarkers included in the study were serum IL-5Ra, nasal IL-5Ra, serum ECP, nasal ECP. The changes in values from baseline were studied in two of the six studies. In particular, reslizumab lead to a statistically significant decrease in all these biomarkers in both treatment groups.<br>Lastly, with the exception of nasal ECP (p=0,26), all of the aforementioned biomarkers were significantly decreased by the use of mepolizumab as compared to placebo.

Authors/researchers/ year of publication	Aim of study/parameters assessed	Type of study	Trials and patients included	Outcomes in brief
Iqbal I et al [18]<br><br>2020	Identification of the role of monoclonal antibodies (Relizumab, Dupilumab, Mepolizumab, Omalizumab) in the treatment of CRS with regard to comparability with current medical treatment, efficacy and risk of complications<br><br>Parameters assessed:<br><br>-Biologic Markers (blood eosinophil count, eosinophilic cationic protein, secreted Il-5a, total serum IgE, Plasma eotaxin-3)<br><br>-Quality of life markers (VAS, SNOT-20, SNOT-22, SF-36, RSOM-31, EQ-5D (EuroQOL 5-Dimension Questionnaire), AQLQ<br><br>-Adverse events	Systematic review	6 RCT's<br>n=243<br><br>-Reslizumab (1), n= 24<br>-Dupilumab (1) , n= 60<br>-Mepolizumab (2), n= 105, 30<br>-Omalizumab (2), n= 24, 14	Omalizumab and mepolizumab demonstrated improvements in Endoscopic Nasal Polyp Score and Symptom score in patients with CRSwNP compared with placebo<br>Dupilumab resulted in 70% reduction in EPS vs 20% in placebo group<br><br>**Reslizumab** induced a sustainable reduction in blood eosinophil count from 12 hours after administration. This returned to baseline at 12 weeks and a rebound increase thereafter, which was >100% in 6 patients from the 1-mg/kg group and in 4 patients from the 3-mg/kg group.<br><br>Further analysis revealed that only those with IL-5 levels of >40 pg/mL in nasal secretions responded to treatment. The authors concluded that IL-5 and eosinophils played a key role (IL-5 dependent) in 50% of the nasal polyps, whereas in the others eosinophilia may have been dependent on other factors (IL-5 independent).<br><br>Serum eosinophilic cationic protein (ECP) and secreted IL-5α decreased with **reslizumab** compared with placebo for the first few weeks after treatment.<br>There was also a reduction in eosinophil count when comparing **dupilumab + MFNS** with placebo + MFNS.<br>This was also statistically significant when measuring total serum IgE and plasma eotaxin-3 levels.<br><br>In contrast, there was no change in plasma or nasal eotaxin levels throughout the study with **reslizumab**.<br><br>In the Bachert et al study, patients receiving **mepolizumab** demonstrated a reduction in blood eosinophil count from baseline to week 25. This was not seen in the placebo group. At week 8 after treatment with **mepolizumab**, the treatment group showed a significant reduction in blood eosinophils.

Authors/researchers/ year of publication	Aim of study/parameters assessed	Type of study	Trials and patients included	Outcomes in brief
Walter Sophie et al [19]<br><br>2020	Assessment of immunological and histological response of airway mucosa to monoclonal antibody therapy (5 agents tested) compared with baseline or a comparison group in patients with respiratory diseases.<br>Parameters assessed:<br>Tissue response was defined by immunological and histological biomarkers such as inflammatory cell number, cell product deposition, cytokine concentration and others	Systematic review	9 RCT's<br>2 Single-arm Trials<br><br>-Omalizumab: 4 RCT's, 1 Trial n= 30, 23, 15, 18 and 9 respectively<br>-Mepolizumab: 2 RCT's, 1 Trial n= 24, 24, 10<br>-Benralizumab: 1 RCT n= 27/13+14<br>-Dupilumab: 1RCT n= 12<br>-Tralokinumab: 1 RCT n= 76	Omalizumab reduced the mucosal concentration of IgE<br><br>Dupilumab reduced IL-13 concentration but not IL-4<br><br>Omalizumab, mepolizumab and benralizumab reduced tissue eosinophil cell density<br><br>Dupilumab reduced mucosal eosinophil granule proteins<br><br>Tralokinumab din not affect the airway mucosa
Peters A T et al [20]<br>2021	Indirect Treatment Comparison of Biologics in Chronic Rhinosinusitis with Nasal Polyps NPS, NC, Loss of Smell, UPSIT, TSS, SNOT-22<br><br>The authors also try to discuss/comment on possible biomarkers to justify the selection or predict the efficacy of biologic agents in CRSwNP	Indirect Treatment Comparison ITC & responder analysis<br><br>Sub-group analyses for asthma and AERD	4 RCT's: n=989<br>SINUS-24: dupilumab vs placebo<br>SINUS-52: dupilumab vs placebo<br>POLYP-1: omalizumab vs placebo<br>POLYP-2: omalizumab vs placebo	Dupilumab had significantly greater improvements from baseline to week 24 vs omalizumab across NPS, loss of smell, UPSIT and TSS. Improvement in SNOT-22 was greater in dupilumab vs omalizumab but not statistically significant<br><br>Currently no baseline biomarker parameters have been identified to select appropriate biologic treatments for individual patients with CRSwNP.<br><br>In the absence of such biomarkers, treatment effect size and responder analysis results may guide the choice of treatment with the greatest improvements in both objective and subjective measures.<br><br>The higher treatment effect size of dupilumab may be due to a broader mechanism of action on type 2 inflammation compared with omalizumab, because dupilumab targets IL-4 and IL-13, which have been shown to decrease IgE production as well as reduce other aspects of type 2 inflammation such as eotaxin-3, IL-5, tissue eosinophilia and mast cell production of inflammatory mediators.

Authors/researchers/ year of publication	Aim of study/parameters assessed	Type of study	Trials and patients included	Outcomes in brief
Gevaert et al [21] 2022	The aim of the review is to provide an overview of eosinophil and IL-5 biology and explore the available evidence relating to the mechanistic role of eosinophils and IL-5 in addition to other type-2 inflammatory mediators in CRSwNP pathogenesis<br><br>The authors also evaluate existing evidence supporting the potential role of eosinophils and IL-5 as biomarkers of NP recurrence following Endoscopic sinus surgery, or as predicting factors for response to biologic treatment (with omalizumab, mepolizumab, dupilumab, benralizumab and reslizumab)	Review	Narrative review,<br>no statistical analysis provided	**Mepolizumab and reslizumab** reduce eosinophil counts, nasal and peripheral IL-5, soluble IL-5Ra and ECP levels in patients with CRSwNP. Mepolizumab also reduces several other local and systemic markers of type-2 inflammation, such as IgE, periostin, nasal matrix metallopeptidase 9 and myeloperoxidase. Mepolizumab decreases numbers of circulating basophils and nasal levels of proinflammatory mediators such as prostaglandin D2, prostaglandin F2 alpha, leucotriene B4 and thomboxane in patients with CRSwNP and N-ERD.<br><br>**Benralizumab** in addition to standard-of-care reduced nasal polyp score, nasal blockage and difficulties in sense of smell along with eosinophil counts in patients with CRSwNP<br><br>**Omalizumab** improves clinical symptoms of CRSwNP and also reduces serum periostin, ECP and soluble IL-5Ra levels but with limited effect on local IL-5 and blood eosinohil counts.<br><br>**Dupilumab** improves clinical symptoms along with decreasing NP levels of CCL24, CCL26, ECP, IL-5, IgE and pulmonary and activation-regulated chemokine.<br>**Dupilumab** is associated with a transient increase in blood eosinophil counts which returns to pre-treatment baseline by 52 weeks post-treatment initiation.<br>Patients with CRSwNP who have higher levels of IL-5 demonstrate greater responses to anti-IL-5 treatment.

Authors/researchers/ year of publication	Aim of study/parameters assessed	Type of study	Trials and patients included	Outcomes in brief
Guo et al [22] 2022	The aim of this review is to summarize the clinical and biological markers associated with poor prognosis of CRS patients under conventional treatment and to provide updated evidence about potential biomarkers for efficacy of T2 biologic agents.	Review	Narrative review, no statistical analysis provided<br><br>Biomarkers related to disease diagnosis and severity were not included	Blood eoshinoplilia and high levels of Charcot-Leyden crystal, cystatin SN, CCL17, MIP-1β and IL-5 in nasal secretions might be good predictors of treatment outcomes (objectivity, nonivasiness, accessibility)<br><br>Blood eosinophil level is a potential biomarker for anti-IL-5 (mepolizumab) and anti-IL-5R (benralizumab) biologic treatment in refractory CRSwNP
Lou et al [23] 2019	The aim of the review is to summarize the known biomarkers and state of endotype-specific treatment strategies and identify future research priorities, including the development of therapies targeting non-type 2 inflammation	Review	Narrative review, no statistical analysis provided	CRSwNP patients with Th2-biased inflammatory responses, eosinophilic polyps, and high serum IgE levels might benefit from the use of biologics.<br>The level of IgE in CRSwNP is associated with eosinophilic inflammation.<br><br>Mepolizumab, benralizumab, and reslizumab could significantly decrease eosinophil counts in peripheral blood.<br>Furthermore, other biomarkers associated with eosinophils (e.g., periostin and ECP) could be suppressed by mepolizumab and omalizumab, suggesting that they might serve as potential biomarkers for the use of biologics.

Authors/researchers/ year of publication	Aim of study/parameters assessed	Type of study	Trials and patients included	Outcomes in brief
Guo et al [24] 2023	This review summarizes the literature data on type-2 biomarkers, with a specific focus on the **indication to biologics** for severe CRSwNP.	Review	Narrative review, no statistical analysis provided	Clinical markers, nasal secretion biomarkers (e.g., eosinophil cationic protein and interleukin-5), blood and nasal cytology eosinophil counts, and nasal swab eosinophil peroxidase activity have been reported to be associated with type 2 inflammation in CRSwNP.<br>The time duration since the last surgery, SNOT-22 score at 1 week of treatment, and baseline serum osteoprotegerin levels might indicate the response to dupilumab.<br><br>LMS and asthma control test scores were found to have moderate predictive value for acceptable improvement after 24-week treatment of omalizumab.<br><br>High blood eosinophil levels at baseline were associated with treatment response to mepolizumab and benralizumab.
Staudacher et al [25] 2020	The aim of the review is to discuss existing biomarkers and potentially new methods that could guide treatment choices in CRS patients	Review	Narrative review, no statistical analysis provided	Numbers of tissue and/or peripheral eosinophils as well as levels of IgE may predict disease severity in CRSwNP but not necessarily treatment responses.<br><br>The majority of CRSwNP patients treated with Dupilumab reported benefit with the drug compared to placebo but this was irrespective of their number of peripheral eosinophils.<br><br>Patients with higher peripheral eosinophil numbers prior to treatment tended to report greater symptom improvement with the drug than those with lower eosinophils numbers. However, no specific numerical cut-off could be established<br><br>Peripheral eosinophils were also not a biomarker of response in studies evaluating Mepolizumab in CRSwNP. In a post hoc analysis, baseline peripheral eosinophil numbers were not predictive of patients who had a 1-point reduction in nasal polyp size.

Authors/researchers/ year of publication	Aim of study/parameters assessed	Type of study	Trials and patients included	Outcomes in brief
Laidlaw et al [26] 2020	The aim of this review is to provide an update on the existing studies of biologics in CRSwNP and to identify potential future areas (including biomarkers) for further research.	Review	Narrative review,<br>no statistical analysis provided	Treatment with omalizumab for 12 months (Hayashi et al 2016) induced significant reductions in urinary levels of leukotriene E4 (LTE4) and the prostaglandin D2 metabolite 9a,11b-prostaglandin F2 (PGD2M), both markers of mast cell activation. In addition, the subjects also had reductions in exacerbations, hospitalizations, systemic corticosteroids, and nasal and asthma symptom scores<br>In a randomized, double-blind, placebo-controlled study of 24 subjects (Gevaert 2006), a single dose of IV reslizumab at 1 mg/kg or 3 mg/kg reduced the total nasal polyp score in half of the subjects receiving reslizumab. A responder analysis showed increased nasal secretion IL-5 levels in responders versus non-responders. Twelve weeks after withdrawal of reslizumab, there was a deterioration in nasal polyp score in responders.<br><br>Laidlaw et al further analyzed the 19 subjects from NCT01920893 who demonstrated aspirin-exacerbated respiratory disease (AERD), a subgroup of CRSwNP patients with comorbid asthma and aspirin intolerance.<br><br>CD45+ CD4- IL-4 producing cells were found to be elevated in patients with AERD and likely reflect the important role of the mast cell in the pathogenesis.<br><br>Future studies focused on biomarker-based endotyping and responder analyses will allow for optimization of personalized treatment for chronic rhinosinusitis with nasal polyp patients.

Authors/researchers/ year of publication	Aim of study/parameters assessed	Type of study	Trials and patients included	Outcomes in brief
Boyle et al [27] 2020	The aim of the review is to describe the basis for using dupilumab in CRSwNP and presents an overview of recent data from three clinical trials which demonstrate the efficacy and safety of dupilumab for CRSwNP treatment<br>The review also contains a few data concerning possible biomarkers (data from NCT01920893 and SINUS-52 trials), which might be used as predictors of the efficacy of the specific biologic	Review	Narrative review, no statistical analysis provided<br><br>3 trials reviewed:<br><br>NCT01920893<br><br>SINUS-24&SINUS-52	Eotaxin-3 and total IgE in the nasal secretions of patients receiving dupilumab decreased significantly compared with those from the placebo group, while the decrease in ECP did not reach statistical significance (Jonstam et al 2019) .<br><br>In the analysis of polyp tissue, total IgE, ECP, eotaxin-2, eotaxin-3, IL-13 and PARC were significantly lower in the dupilumab group by the end of treatment than in the placebo group, whereas IL-6, IL-1β, IL-4, eotaxin-1, IL-5, IL-10, IL-17, IL-33, TNF-α or TARC were not significantly different between groups at the end of treatment<br><br>Measurement of nasal ECP, eotaxin-3 and total IgE were unique to the SINUS-52 protocol. Local and systemic biomarkers of inflammation showed significant improvements with use of dupilumab when compared with placebo
Wang et al [28] 2019	This review provides an up-to-date summary of emerging studies of biologics for CRSwNP and describes current trends as well as future perspectives for clinical practice.<br><br>The authors state that it is necessary to identify effective biomarkers in the blood, nasal secretion, and biopsy samples.	Review	Narrative review, no statistical analysis provided	Patients with CRSwNP and asthma whose levels of IgE in the serum are at least 30 IU/ml can be treated with omalizumab. However, patients with IgE levels less than 76 IU/ml do not benefit significantly. Local IgE is associated with an inflammatory state of the tissue and recurrence of polyps.<br>Mepolizumab, benralizumab, and reslizumab target and decrease eosinophil counts. Mepolizumab shows better efficacy for reducing both blood and tissue eosinophils, whereas dupilumab reduces tissue eosinophils Additionally, although dupilumab shows anti-type 2 inflammation activity, it cannot significantly change the eosinophil count in the serum.<br>Gevaert et al. (2006) found that patients showing greater than 40 pg/ml increases in IL-5 in their nasal secretions were more suitable for treatment with anti-IL-5.<br><br>Dupilumab can block the IL-4Ra subunit to inhibit the production of IL-4 and IL-13. Therefore, IL-4 or L-13 can be used as a biomarker for dupilumab treatment.<br><br>Wang et al. found that the expression of periostin is related to IL-4 and IL-13 rather than to IL-5. Jonstam et al. showed that IL-5 is positive in tissues when the level of periostin in the serum is higher than 48.5 ng/ml (sensitivity 93.5%, specificity 62.5%). Periostin was confirmed to be decreased after treatment with mepolizumab and omalizumab.<br><br>Decrease of ECP in serum or secretion was found after reslizumab and mepolizumab therapy.<br><br>Other biomarkers, such as IL-25 and IL-33 have also been evaluated. Combining biomarkers may have greater effects than using one biomarker.<br>The exact cut-off values of biomarkers for predicting the response to biologics must be further evaluated.

Authors/researchers/ year of publication	Aim of study/parameters assessed	Type of study	Trials and patients included	Outcomes in brief
Pauwels et al [29] 2015	This review highlights the biologic agents that might be beneficial as adjunctive therapy in CRSwNP uncontrolled by current standard treatment.<br><br>The authors also comment on (expert opinion session) possible biomarkers which might help in patients and/or biologic agent selection for maximizing the treatment effect in CRSwNP	Review	Narrative review, no statistical analysis provided	CRSwNP patients with asthma, recurrence of polyps after surgery and high levels of serum IgE, possibly reflecting even higher IgE concentrations in the polyp tissue, would be a plausible group for treatment with omalizumab.<br><br>Patients who were good responders to reslizumab had increased IL-5 levels in nasal secretions at baseline, so this might be a biomarker to use in finding patients suitable for this treatment.<br><br>Benralizumab might be interesting to further investigate as treatment for CRSwNP because it also decreases basophil levels, which were shown to be elevated in nasal polyps and play an important role in the allergic response.<br><br>Treatment with biologics against either IL-4 or IL-13 has not shown convincing benefits in clinical outcome. This might suggest that blocking both cytokines is required to obtain a more complete inhibition of this Th2 inflammatory pathway.<br><br>Dupilumab was the only mAb targeting the common IL-4/IL-13 pathway investigated in CRSwNP to date (by the time of publication of this specific review).
